# Ectoparasitism and the gut microbiota: a cross-fostering experiment in a wild passerine

**DOI:** 10.1093/femsec/fiag079

**Published:** 2026-07-17

**Authors:** Antonio José García-Núñez, Juan José Soler, Jordi Figuerola, Libya González-Dávalos, José Antonio Morillo, Cristina Ruiz-Castellano, B Irene Tieleman, Manuel Martínez-Bueno, Gustavo Tomás

**Affiliations:** Departamento de Ecología Funcional y Evolutiva, Estación Experimental de Zonas Áridas (CSIC), 04120 Almería, Spain; Departamento de Ecología Funcional y Evolutiva, Estación Experimental de Zonas Áridas (CSIC), 04120 Almería, Spain; Unidad Asociada (CSIC): Coevolución: cucos, hospedadores y bacterias simbiontes, Universidad de Granada, 18071 Granada, Spain; Departamento de Biología de la Conservación y Cambio Global, Estación Biológica de Doñana (CSIC), 41092 Sevilla, Spain; Centro de Investigación Biomédica en Red Epidemiología y Salud Pública (CIBERESP), 28029 Madrid, Spain; Departamento de Ecología Funcional y Evolutiva, Estación Experimental de Zonas Áridas (CSIC), 04120 Almería, Spain; Departamento de Ecología Funcional y Evolutiva, Estación Experimental de Zonas Áridas (CSIC), 04120 Almería, Spain; Departamento de Ecología Funcional y Evolutiva, Estación Experimental de Zonas Áridas (CSIC), 04120 Almería, Spain; Groningen Institute for Evolutionary Life Sciences (GELIFES), University of Groningen, Nijenborgh 7, 9747 AG Groningen, The Netherlands; Unidad Asociada (CSIC): Coevolución: cucos, hospedadores y bacterias simbiontes, Universidad de Granada, 18071 Granada, Spain; Departamento de Microbiología, Universidad de Granada, 18071 Granada, Spain; Departamento de Ecología Funcional y Evolutiva, Estación Experimental de Zonas Áridas (CSIC), 04120 Almería, Spain; Unidad Asociada (CSIC): Coevolución: cucos, hospedadores y bacterias simbiontes, Universidad de Granada, 18071 Granada, Spain

**Keywords:** Carnus hemapterus, gut bacterial symbionts, nestling growth, nestling relatedness, phenotypic condition, spotless starlings

## Abstract

The gut microbiota plays a key role in host development, physiology, and immunity, and is likely shaped by hosts’ genetic and environmental factors, including ectoparasitism and, thus, host health. Here, we experimentally manipulated ectoparasite (*Carnus hemapterus*) abundance in spotless starling (*Sturnus unicolor*) nests. We evaluated its hypothesized disruptive effects on nestling gut microbiota and explored also whether the effects depend on sibling relatedness using a partial cross-fostering design. Experimentally increased parasitism significantly affected alpha but not beta diversity of the bacterial community in 14-day-old cross-fostered nestlings, i.e. those non-genetically related to their social parents and siblings. The experimental parasite manipulation also affected the relative abundance of 123 bacterial strains, including fermenters, mucosa-adapted bacterial symbionts, and opportunistic pathogens. Finally, gut microbiota composition was also associated with nestling phenotype (body mass, wing and tarsus length, carotenoid concentration, and telomere dynamics). Sparse PLS network analyses linked body mass (positively: *Erysipelatoclostridium, Parabacteroides*, and *Helicobacter*; negatively: *Salmonella*) and parasitism intensity (negatively: *Dubosiella, Rickettsiella*, and *Helicobacter*) with relative abundance of specific bacterial strains. These results provide the first evidence in natural conditions that the effects of ectoparasites on gut microbiota depend on genetic relatedness between nestlings and parents, highlighting the complex interplay between parasitism, microbiota, and host traits.

## Teaser

Contribution to understanding how early-life parasitism shapes gut microbiota diversity and composition in a wild bird species, and how these effects depend on genetic relatedness between nestlings and their parents.

## Introduction

The gut microbiota is a complex community of microorganisms residing within the intestines of their animal hosts (Hasan and Yang 2019). It plays a crucial role in host health by contributing to essential processes such as nutrient metabolism (Rowland et al. 2018), immune system modulation (Sanchez et al. 2015), and the maintenance of gut barrier integrity (Chelakkot et al. 2018). The composition and functionality of the gut microbiota is, at least partially, influenced by a complex interplay of host factors, including sex, age, gut region, genetic background, and behavioural and environmental factors (Grond et al. 2018, Lee et al. 2020, Matheen et al. 2022). Among the environmental factors affecting the composition and diversity of the animal gut microbiota, diet is by far the best-known environmental driver (Schmiedova et al. 2022). The association between microbiota and other factors such as geographic location (Pin Viso et al. 2021), exposure to pollutants (Bariod et al. 2025), urbanization (Solomon et al. 2023), and parasitism (Li et al. 2023), has garnered significant attention among the scientific community in recent years. For instance, by activating the immune system of their hosts, some parasites are indirectly responsible for maintaining homeostasis within the gut microbiota (Partida-Rodriguez et al. 2017) and, thus, for the diversity, composition, and functionality of the symbiotic community (Li et al. 2023). Crucially, parasite-induced perturbations of the gut community can favour the expansion and/or the emergence of strains with pathogenic potential, as documented for both endoparasites and ectoparasites (Swe et al. 2014, Beatty et al. 2017, Reynolds et al. 2017, Wang et al. 2024). In accordance, Wang et al. (2024) examined the impact of ectoparasitic flea infestations on the gut microbiota of *Meriones unguiculatus* (gerbils), finding that parasite presence significantly increases the abundance of harmful strains and altered immune response and both diversity and composition of the microbiota.

Detrimental effects of parasites (Price 1980) can be reflected in the gut microbiota (Videvall et al. 2020, Worsley et al. 2021). These associations might not only be the consequence of parasites consuming host resources, which would affect gut environment in terms of resource availability, but also by their direct and indirect effect on host microbiota. In the case of blood-feeding ectoparasites, such effects can arise through blood loss, skin damage, immune activation, and physiological stress, which alter host nutritional state and gut conditions and, thereby, may favour changes in microbial diversity and in the relative abundance of opportunistic bacteria (Hoi et al. 2018, Wang et al. 2024). Moreover, via gut–brain axis communication, the gut microbiota could also influence phenotypic condition, immune response, and behaviour of their hosts (Guernier et al. 2017, Sherwin et al. 2019) and, thus, susceptibility to parasitic infections. Accordingly, variation in gut microbiota has been linked to physiological markers of host condition, including circulating carotenoids and telomere dynamics (Velando et al. 2021, Eroglu et al. 2023). In other words, gut microbiota characteristics might be a consequence of parasitism, but the risk of parasitism (i.e. host defences) might also be affected by the gut microbiota. This bidirectional relationship may have important evolutionary implications, because parasite-induced changes in the gut microbiota could affect host growth, immune performance, and stress physiology, thereby influencing survival prospects and the expression of defensive resistance or tolerance to parasitism. This scenario therefore highlights (i) the importance of considering parasites when studying the association between phenotypic quality and the gut microbial communities of their hosts, and (ii) that experimental approaches are essential to discern between causes and consequences of associations between the microbiota and host parasitism, immunity, and phenotype. However, with few exceptions (Wang et al. 2024), most studies examining the association between parasitism and the gut microbiota are correlational (Matheen et al. 2022) and focused on endoparasites and mammals (Marsh et al. 2024).

In addition, both gut microbiota (Lee et al. 2020) and immune response to parasitism (Sorci et al. 1997, Brinkhof et al. 1999) have a significant host genetic component. Thus, nestlings may differ in their susceptibility to parasite-induced microbiota changes depending on genetic relatedness between parents and offspring, possibly because parents may invest differently in related and non-related nestlings, as suggested by experimental evidence from the brood parasitism literature (Soler et al. 2014, Soler, M. et al. 2017). Moreover, gut microbiota may even depend on pre- and post-hatching maternal effects. Through prenatal maternal effects (e.g. deposition of hormones, immune factors, or antibodies in the eggs), mothers may even prepare offspring for expected risk of parasitism (Groothuis and Schwabl 2008, Hasselquist and Nilsson 2009, Moreau et al. 2022), which may directly or indirectly affect the gut microbiome of their offspring. In addition, maternal microbes can be transferred directly to the embryonic gut during egg formation (Gong et al. 2023). Postnatal parental effects may also be important. Level of parental care, including nest sanitation and parasite removal, may also vary among reproducing animals (Hurtrez‐Boussès et al. 2003) and be adjusted to genetic relatedness between parents and offspring (Møller and Birkhead 1993, Neff 2003), which will further predict an indirect genetic effect on the gut microbiota of developing offspring. Then, considering the genetic relatedness between parents and nestlings, based on comparing genetically related and social (cross-fostered) siblings, might also help to identify the hypothesized effect of parasitism on the gut microbiota of animals, a possibility that as far as we know, has never been considered.

Field-based microbiome studies that examine the factors influencing the gut microbiota are relatively scarce for wild birds compared to mammals (Sun et al. 2022) or poultry (Hird 2017, Matheen et al. 2022). To date, only two studies have approached the issue experimentally and, despite some limitations, have found support for a direct effect of ectoparasites on the diversity and composition of the intestinal microbiota of wild birds (Ingala et al. 2021, Solomon et al. 2023). Both studies used permethrin to manipulate the natural abundance of parasites within avian nests (Kleindorfer and Dudaniec 2016), but this insecticide may also have negative effects on developing nestlings (Bulgarella et al. 2020, Tassin de Montaigu et al. 2025) and on their gut microbiota (Nasuti et al. 2016). Thus, the possibility that the detected effects were an undesired consequence of permethrin treatment rather than the effect of the reduced parasitism cannot be ruled out, therefore, direct manipulation of parasitism is necessary to reach a more robust conclusion.

Here, we manipulated the parasite population density by adding *Carnus hemapterus* (hereafter Carnus) (Liker et al. 2001) adult flies to spotless starling (*Sturnus unicolor*) nests, and explored the effects of experimentally increased parasitism on (i) the diversity and composition of the gut microbiota and (ii) nestling phenotype (i.e. body mass, carotenoid concentration, and telomere dynamics). Moreover, we explored the associations between phenotypic condition and microbial alpha and beta diversity, as well as the relative abundance of specific bacterial strains. In addition, to isolate the effect of experimentally increased parasitism while controlling for possible pre-hatching parental effects and/or genetic relatedness, we performed a partial cross-fostering experiment exchanging hatchlings between nests. Then, when exploring the effects of experimental infestation on the gut microbiota of nestlings, we considered whether experimental nestlings were genetically related or not to the parents where they grew up (i.e. own or foster offspring). Such associations were explored in sibling nestlings at two different developmental stages that were or were not genetically related, which allowed to disentangle the effect of age and genetic relatedness.

## Methods

### Study area and species

Fieldwork was carried out during the 2021 breeding season (March–June) in a population of spotless starlings (hereafter starlings) situated in southern Spain, at the old railway station of La Calahorra (37°15′N, 3°01′W). This area, located on a high-altitude plateau in the semiarid Hoya de Guadix, has ca. 100 cork-made nest boxes attached to tree trunks or walls that are used by starlings for breeding.

The starling is a medium-sized, hole-nesting altricial passerine. In our study population, starlings start egg-laying by mid-April, typically producing clutches of 4–5 eggs, with one egg laid per day. Incubation is predominantly carried out by the female and typically begins one day before the last egg is laid, spanning a duration of 11 days. The nestling period lasts ∼18 days but can extend up to 25 days (Veiga and Polo 2016, Soler, J. et al. 2017). Starling nestlings and adults are commonly parasitized by Carnus (Avilés et al. 2009, López-Rull et al. 2010, Tomás et al. 2018, Azcárate-García et al. 2020), a blood-sucking fly that sheds its wings after locating an appropriate active nest (Grimaldi 1997), that negatively affects nestling body condition and immune response (Hoi et al. 2018). Thus, our research system and experimental approach are appropriate for the aims of the study.

### Fieldwork and cross-fostering experiment

From April to June, nest boxes in the area were monitored every three days to identify the laying date of the first egg and, then, every other day until the clutch was complete. Twelve days after laying, we began daily nest visits, continuing until hatching. On the second day after the first nestling hatched, nests were randomly assigned to one of two treatments. Experimental nests received 10–15 unwinged *Carnus* flies collected from nearby nests, while control nests were visited without the addition of ectoparasites (see Tomás et al. 2018). Before experiment application, a partial cross-fostering experiment was conducted, exchanging two nestlings between control and experimental nests paired according to hatching date and brood size. This pairing allows comparing own and foster siblings that are reared in the same nest and by the same parents.

At the time of nestling cross-fostering, all nestlings within each nest were weighed using a digital balance (Kern, Germany, precision 0.1 g) and identified by clipping part of their downy feathers on their head, back or wings. In half of the paired nests (randomly selected), the first and third nestlings in the body mass hierarchy were cross-fostered, while in the other half of paired nests, the second and fourth nestlings were cross-fostered. Moreover, a drop of blood (ca. 5 μl) was collected from each nestling by brachial venipuncture with the aid of a needle and a 75 μl heparinized capillary tube, which was stored in an Eppendorf tube with absolute ethanol and maintained at 4°C until DNA isolation for estimating telomere length.

During the second nest visit on day 8 post-hatching, cloacal samples were collected from all nestlings in the nest following established protocols (Lee et al. 2020) consisting of injecting and repipetting 500 μl of sterile phosphate buffer (0.1 M Na2HPO4 and 0.1 M NaH2PO4, pH 7.4) in nestlings cloacae using sterile tips and an automatic micropipette. The sampling was performed wearing new latex gloves cleaned with 70% ethanol to avoid between-nests contamination and to approach sterility. Collected samples were stored in 1 ml microfuge tubes containing 500 μl of lysis buffer (50 mM Tris-HCl, 0,5% SDS, 2 mM EDTA, 100 mM NaCl) and kept at 4°C in a portable fridge until transported to the laboratory, where they were stored at −20°C for further analysis. During this visit, all nestlings in the nest were ringed and their body mass was recorded.

On day 14 after hatching, cloacal samples were collected again from all nestlings, as detailed before. Moreover, 300 μl of blood was collected at this age, of which a small fraction was stored in absolute ethanol for estimating telomere length as before, and the rest was kept for estimating plasma carotenoid concentration (see below). Additionally, tarsus length (using a digital caliper with 0.01 mm precision), wing length (using a ruler with 1 mm precision) and body mass of all nestlings were recorded.

A total of 256 nestlings from 83 nests (39 control and 44 experimental) were sampled on day 8, and 210 nestlings from 73 nests (36 control and 37 experimental) on day 14. During each visit, parasitism intensity was assessed by directly counting the number of droppings and/or bites (i.e. spots) on their skin surface (belly and left wing). These spots are reliable indicators of *Carnus* ectoparasite load (López-Rull et al. 2007).

### Laboratory work

#### Estimating blood carotenoid concentration

Blood samples were centrifuged in the field at 18 000 × g RCF for 5 min to separate the plasma from the cells. The plasma was initially stored at –20°C for up to one week before being transferred to –80°C for preservation during a few months until analysis. Carotenoid concentration in blood plasma was quantified using a spectrophotometric assay (Bertrand et al. 2006). In brief, 15 µl of plasma was mixed with 135 µl of ethanol, vortexed and centrifuged at 4°C at 1500 × g RCF for 10 min. We analyzed the supernatant using a spectrophotometer (Sunrise-basic Tecan, 16039400) and measured the optical density of the carotenoid peak at 450 nm. A calibration curve was generated using lutein (CAYM10010811-1, VWR) with absorbance values ranging from 0 to 200 μg × ml–1 (R² = 0.999), allowing us to extrapolate lutein concentrations as a proxy for carotenoid levels in blood plasma.

#### Telomere length estimations

We extracted DNA from blood samples following a standard chloroform-isoamyl alcohol-based protocol (Ferraguti et al. 2013, Soler et al. 2015). DNA concentration was adjusted to 20 ng/µl with distilled water, and samples were stored at –20°C until further analysis. Telomere length was estimated by RT-PCR measuring the relative quantity of telomere sequences to a single-copy reference gene (GAPDH) following Criscuolo et al. (2009). Briefly, each RT-PCR reaction was done in a final volume of 20 µl, containing 10 µl of LightCycler 480 SYBR Green I Master (Roche) and 1 µl of DNA at 20 ng/µl. Due to different PCR conditions, telomere and GAPDH reactions were conducted separately in different plates using a LightCycler 480 RT-PCR System (Roche). Telomere amplification conditions included an initial denaturation at 95°C for 10 min, followed by 30 cycles of 1 min at 56°C and 1 min at 95°C. GAPDH amplification started with 10 min at 95°C, followed by 40 cycles of 1 min at 60°C and 1 min at 95°C. Each sample was run in duplicate, and those with a coefficient of variation above 5% were excluded from the analyses. To generate standard curves, each 96-well plate included serial DNA dilutions (40 ng, 10 ng, 2.5 ng, 0.66 ng per well) from a reference pool, along with a no-DNA control, all run in triplicate. Quantification cycle (Ct) values were transformed into normalized relative quantities (NRQs) following Hellemans et al. (2007), ensuring accurate amplification efficiency control for each RT-PCR reaction. Amplification efficiencies ranged from 1.964 to 2.006 for telomere products and from 2.047 to 2.090 for GAPDH products, while the calibration curve slopes varied between −3.411 and −3.308 for telomeres and between −3.213 and −3.124 for GAPDH. Melting curve analyses confirmed the absence of primer dimers or nonspecific amplifications.

#### DNA extraction, amplicon sequencing, and data processing

DNA was extracted from 600 µl of each cloacal sample containing lysis buffer. Samples were further disrupted by mechanically shock, and DNA was extracted using MSOP (Martín-Platero et al. 2007, Purswani et al. 2011). The V6-V8 regions of the 16S rRNA gene were amplified by PCR from a subset of samples using primers B969F (ACGCGHNRAACCTTACC) and BA1406R (ACGGGCRGTGWGTRCAA) (Comeau et al. 2011). PCR products were visualized on a 1% agarose gel. After confirming the presence of 16S rRNA, samples were submitted for next-generation sequencing (NGS)-based 16S amplicon sequencing on an Illumina NovaSeq (PE250-Seq; Novogene Company Ltd. Cambridge, UK) following the provider's protocols.

#### Bioinformatics

Sequences were processed in QIIME 2 (amplicon-2024.5) (Bolyen et al. 2019). Primer sequences were trimmed with the q2-cutadapt plugin: demultiplexed reads were imported via manifest files and adapter/primer sequences were removed with cutadapt trim-paired command. Denoising was performed per sequencing run with DADA2 (Callahan et al. 2016) using the denoise-paired plugin with paired-end mode, no 5′ trimming (–p-trim-left-f/r 0), truncation both reads at 220 bp (–p-trunc-len-f/r 220) according to quality profiles, and using pseudo-pooling (–p-pooling-method pseudo). Results feature tables and representative sequences from individual runs were then merged. To identify and remove possible contaminants, we used QIIME 2 quality-control decontam plugin with the prevalence method (Davis et al. 2018), incorporating negative controls specified in the sample metadata. Taxonomic assignment of ASVs was performed with the QIIME 2 Naïve Bayes classifier (Bokulich et al. 2018) trained on SILVA 138.1 (Quast et al. 2013) for the 969F–1406R (V6–V8) region; non-target lineages (Eukaryota, mitochondria, chloroplasts) and features lacking phylum-level classification were removed. A phylogenetic tree for downstream diversity analysis was generated with align-to-tree-mafft-fasttree pipeline. Final feature tables, taxonomy, representative sequences, and trees were exported for downstream analyses in R.

### Statistical analysis

Before the analyses, some variables were transformed to meet model assumptions and reduce the influence of extreme values (Zuur et al. 2010, Knief and Forstmeier 2021): *Carnus* dropping counts (hereafter intensity of parasitism) recorded on days 8 and 14 were log-transformed to reduce skewness caused by extreme observations, while telomere length values measured on days 2 and 14 were log-transformed to meet normality requirements (Marasco et al. 2021). Telomere shortening was calculated as the difference in transformed values of telomere length between day 2 and day 14. After these transformations, all model residuals conformed to the assumptions of normality (Kolmogorov–Smirnov tests, *P* > 0.05) and homogeneity of variances (Levene’s tests, *P* > 0.05). In order to minimize the influence of sequencing depth on diversity measurements and ensure equal sequence counts across samples, the ASV table was rarefied to the minimum sampling depth of 23 000 reads using the “rarify_samples” class of the *microeco* package in R environment (McKnight et al. 2018, Liu et al. 2021). To characterize bacterial alpha diversity, we estimated three indices: (i) Richness (the observed ASVs), (ii) the Shannon index, and (iii) the Faith’s phylogenetic diversity index (PD Faith) (Xia and Sun 2023). Between-sample (beta) diversity was quantified using Bray–Curtis (Bray and Curtis 1957), Weighted and Unweighted Unifrac distance matrices (Lozupone and Knight 2005, Lozupone et al. 2007). All diversity indexes were calculated with the *microeco* package (Liu et al. 2021) in R (R Core Team 2025) environment. For beta diversity analyses, the dataset was filtered to reduce the influence of extremely rare taxa, retaining bacterial ASVs within the top 0.008% of relative abundance and with a minimum prevalence of four samples (Cao et al. 2021, Risely et al. 2021, Custer et al. 2023). Hatching date and brood size do not vary within nests, so we followed a residualization approach (Frisch and Waugh 1933, Lovell 2008). Thus, we first ran multiple linear regressions with the focal response of each analysis as the dependent variable and hatching date and brood size as predictor factors (Electronic Supplementary Material (ESM), Table S1, S2, S3), then used the residuals as responses in subsequent analyses (e.g. LMMs, perMANOVAs) that included nest identity as a random effect. The nest term was excluded from the model if its effect on the dependent variable was far from statistical significance (i.e. *P* > 0.25). The LMMs were run in STATISTICA software (Dell-Inc 2015) and perMANOVAs analyses (Anderson 2001, Little et al. 2024) were run in software PRIMER7 v. 7.0.17 (PRIMER-e) (Clarke and Gorley 2015) with the PERMANOVA + add-on (Anderson 2008). Finally, some nestlings died between the first and second sampling (8- and 14-day after hatching) and, thus, to maximize sample sizes, analyses were separately conducted for the two developmental stages.

#### Testing the effects of experimental treatment

First, we tested the assumption that nestlings in experimental nests should be more heavily parasitized and of lower body mass [main predictor of survival; Moreno et al. (2005)] than nestlings in control nests after controlling for the effect of sibling relatedness (own vs cross-fostered) and the random effect of nest identity. To do so, we ran two Linear Mixed Models (LMM) that included experimental treatment and sibling relatedness (own or cross-fostered) as fixed factors, while nest identity nested within experimental treatment was included as the random factor. In the first LMM the response variable was intensity of parasitism and in the second LMM, the response variable was body mass. The interaction between fixed effects was tested in separate models that also included the main effects. Fisher’s LSD test was used for post-hoc pairwise comparisons. Different reproductive events within the same nest-box were considered as independent information because they involved distinct breeding attempts that may differ in parental identity, brood composition, and environmental conditions.

The effects of experimentally increased parasitism on bacterial diversity were tested in LMMs (alpha diversity) and perMANOVAs (beta diversity) that also included sibling relatedness as fixed effect, and nest identity nested within experimental treatment as a random factor. The interaction between fixed effects was tested in separate models that also included the main effects.

To explore whether the experimental treatment influenced the abundance of specific bacterial taxa (i.e. microbiota composition) we used an Analysis of Compositions of Microbiomes with Bias Correction 2 algorithm (ANCOM-BC2, version 2.2.0) (Lin and Peddada 2020) as implemented in the ANCOMBC R package (v2.2.0; function ancombc2) and run through the trans_diff workflow in *microeco* (Liu et al. 2021). This method corrects compositional bias, controls the false discovery rate using the Benjamini–Hochberg procedure, and detects structural zeros for more reliable results. Following differential abundance testing, ASVs identified by ANCOM-BC2 were checked in the NCBI Pathogen Detection portal (National Center for Biotechnology Information 2016) and the FAPROTAX database (Louca et al. 2016) to determine whether any species within the corresponding genera have been catalogued as pathogenic.

#### Association between parasitism, bacterial communities, and nestling phenotypes

To test the association between bacterial alpha or beta diversity and intensity of parasitism, we ran LMM and perMANOVAs models, respectively, that included sibling relatedness and intensity of parasitism as fixed effects and nest identity as random factor. Separate models were run for intensity of parasitism estimated at different ages (day 8 and 14) including the same variables.

To search for specific bacterial strains related to nestling phenotypic traits, sparse Partial Least Squares (sPLS) models in regression mode were used, which effectively reduces dimensionality while addressing collinearity between variables (Chun and Keleş 2010). Phenotypic traits were standardized (mean = 0, SD = 1) to ensure comparability and avoid scale-driven bias in sPLS, improving model stability and variable selection (Abdi 2010, Rohart et al. 2017). Before fitting the final sPLS models, a tuning procedure (tune.spls function) with 50 × 10-Mfold cross validation was applied to extract the phenotypic traits that associate with specific bacterial strains (i.e. ASV). As dependent variables (keepY), we considered the seven phenotypic variables (body mass at day 8 and 14, wing length, tarsus length, carotenoid concentration, telomere length and telomere dynamics). As possible independent factors (keep X), we used CLR-transformed relative abundances of the filtered dataset (see above). Model parameterization (perf function) was used to select the number of latent components to use in our final models (see ESM, Appendix S1). Final model performance was assessed using 10-Mfold cross-validation repeated 50 times. All these analyses were performed with the “mixOmics” v. 6.22.0 package (Rohart et al. 2017) in R. Analogous sPLS analysis (and tuning procedure; ESM Appendix S1) were used to identify bacterial ASVs that associated with intensity of parasitism (keepY) and the same set of CLR-transformed microbiome data as independent variables (keepX).

We also explored the association between gut microbiota and nestling phenotype, for both alpha and beta diversity. For alpha diversity, we used LMMs that included phenotypic information as predictors, alpha diversity indices, and sibling relatedness as fixed factors, and nest identity as the random factor. For bacterial beta diversity, Euclidean distance matrices of differences in phenotypic traits (calculated in “*vegan*” package (Oksanen et al. 2022) were used as dependent matrices in separate Multiple Regression on distance Matrices (MRM) that were run using the “ecodist” v. 2.0.9 package (Goslee and Urban 2007) within R environment to estimate partial correlation coefficients after controlling for other factors of interest. MRM included the following independent matrices: (i) a beta diversity matrix of the bacterial communities, (ii) a binary matrix indicating nest identity (1 or 0 values for nestlings that do or do not share the same nest box), (iii) a binary matrix for nestling relatedness (1 or 0 values for own and foster nestlings, respectively), and matrices of differences in (iv) hatching date and (v) brood size.

## Results

### Experimental effects on intensity of parasitism and body mass

**Figure 1:**
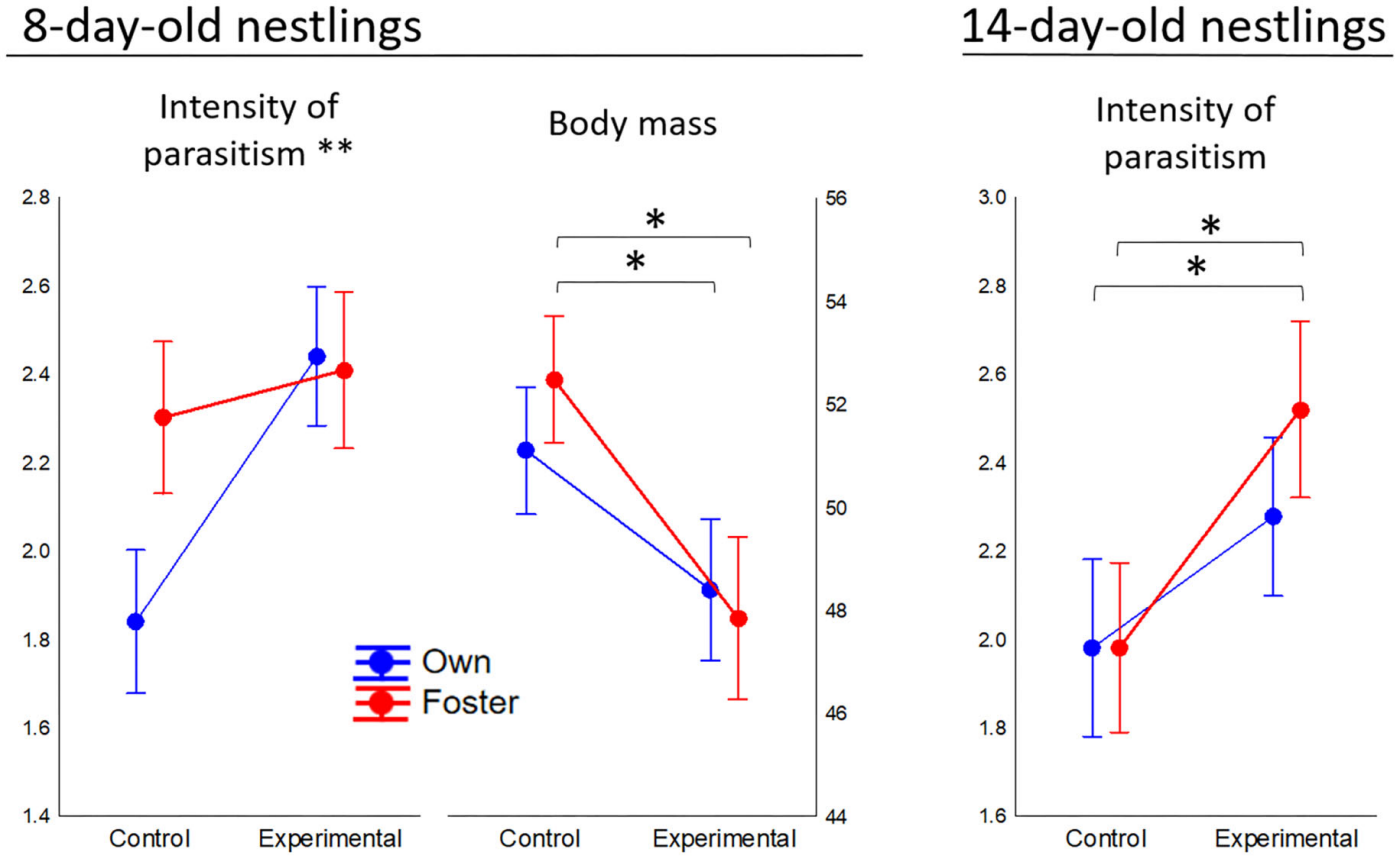
Weighted means (± SE) of intensity of parasitism at day 8 and 14 and body mass at day 8 for experimental and control starling nestlings from different origin (residuals after controlling for the effects of brood size and hatching date, see text). An asterisk next to the panel title indicates that the interaction term in the model was significant. Asterisks above brackets indicate significant post-hoc comparisons: * *P* < 0.05, ** *P* < 0.01, *** *P* < 0.001.

Experimental treatment had a significant effect on intensity of parasitism experienced by 8-day-old nestlings in interaction with sibling relatedness (Table 1). Nestlings in experimental nests showed a higher intensity of parasitism than those in control nests, but only among nestlings that remained in their nest of origin (F_1,81_ = 7.35, *P* = 0.007) (Fig. 1). At day 14, neither experimental treatment, sibling relatedness nor their interaction significantly affected intensity of parasitism (F_1,71_ = 1.68, *P* > 0.19) (Table 1). However, Fisher’s LSD post hoc tests showed that experimental–fostered nestlings were exposed to significantly higher intensity of parasitism than both control–own and control–fostered nestlings (both *P* = 0.017) 14 days after hatching (Fig. 1).

**Table 1:** Results from linear mixed models testing the effects of experimental treatment and sibling relatedness (own vs. foster nestlings) on residuals of intensity of parasitism and residuals of body mass, for 8 and 14-day-old starling nestlings.

	Intensity of parasitism day 8		Intensity of parasitism day 14		Body mass day 8		Body mass day 14	
Predictors	F_1,81_	*P-value*	F_1,71_	*P-value*	F_1,81_	*P-value*	F_1,71_	*P-value*
Intercept	0.03	0.87	0.08	0.78	0.00	0.97	0.01	0.92
Treatment	0.95	0.33	1.48	0.23	3.22	0.08	0.61	0.44
Sibling relatedness	1.03	0.31	0.43	0.51	0.15	0.70	0.87	0.35
Treatment * Sibling relatedness	7.37	**0.007**	1.75	0.19	1.09	0.30	0.03	0.87
Nest ID (treatment)	3.37	**<.001**	2.78	**<.001**	1.59	**0.01**	2.18	**<.001**
Intercept	0.10	0.75	257.80	**<.001**	0.10	0.75	257.80	**<.001**
Treatment	0.70	0.40	1.99	0.16	0.70	0.40	1.99	0.16
Sibling relatedness	1.19	0.28	0.45	0.50	1.19	0.28	0.45	0.50
Treatment * Sibling relatedness	7.37	**0.007**	1.67	0.20	7.37	**0.007**	1.67	0.20
Nest ID (treatment)	3.54	**<.001**	3.10	**<.001**	3.54	**<.001**	3.10	**<.001**

Nest identity nested within experimental treatment was included as a random factor. Interaction effects were tested in separate models that also included the main effects. Values in bold indicate significance at α < 0.05.

Regarding body mass, although neither experimental treatment, sibling relatedness, nor their interaction showed significant effects on this trait for 8 and 14-day-old nestlings (Table 1), Fisher’s LSD post hoc test showed that both own and fostered 8-day-old nestlings in experimental nests were heavier than those in control nests (*P* < 0.02, Fig. 1).

### Gut microbiota of starling nestlings

**Figure 2:**
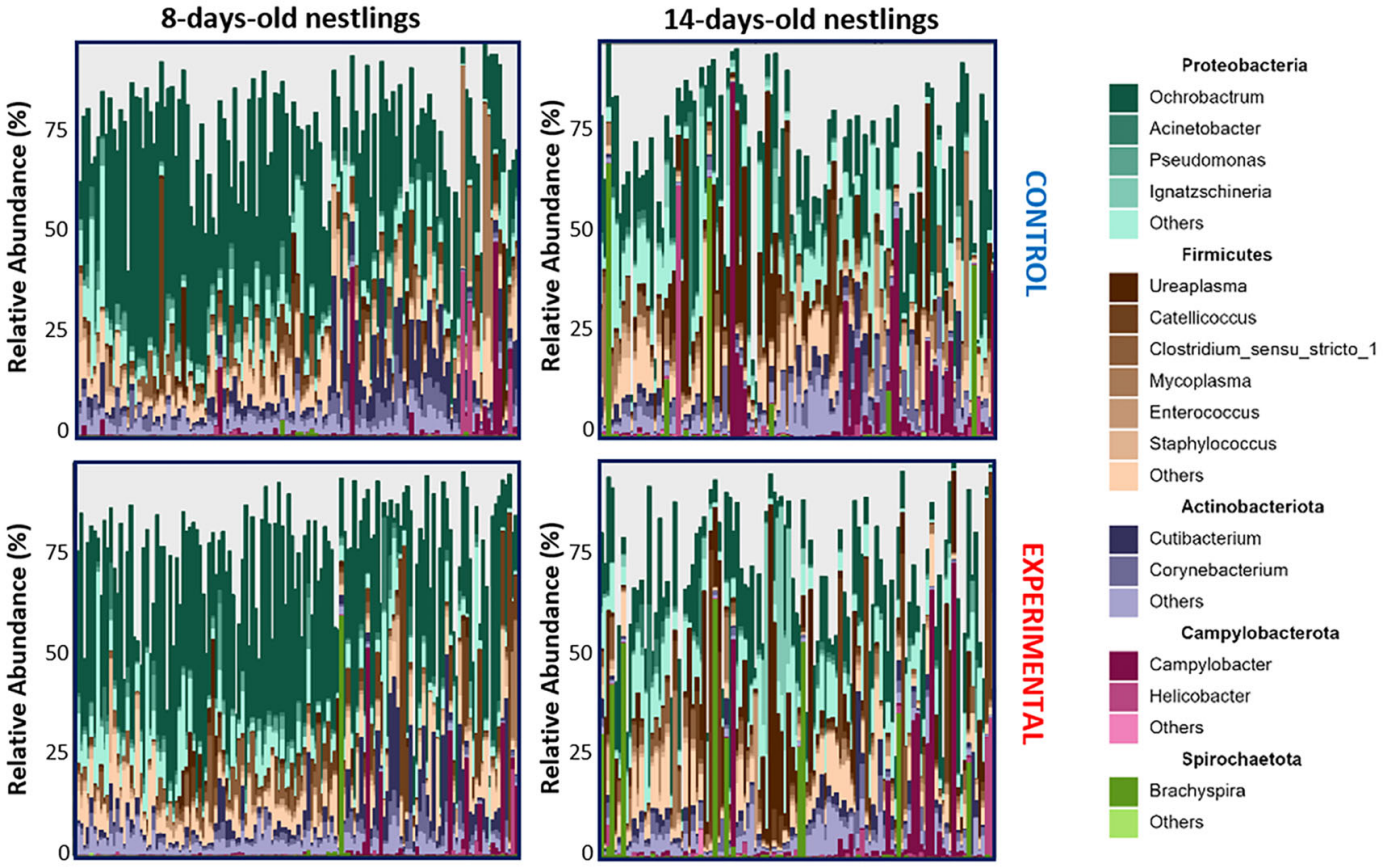
Stacked barplot showing the relative abundance of the most frequently detected genera for each individual starling nestling. The figure provides information on treatment (control versus experimental) and day of sampling (8 versus 14).

Illumina analyses resulted in 31 698 239 reads that were classified into 28 166 ASVs. After rarefaction, 9 840 056 reads were retained, corresponding to 28 010 ASVs. These ASVs were classified within 45 phyla. The bacterial community was largely dominated by Proteobacteria (58.8%), Firmicutes (22.1%), Actinobacteriota (9.4%), Campylobacterota (3.53%), and Spirochaetota (1.42%) (Fig. 2).

### Microbial bacterial communities and parasitism

**Figure 3:**
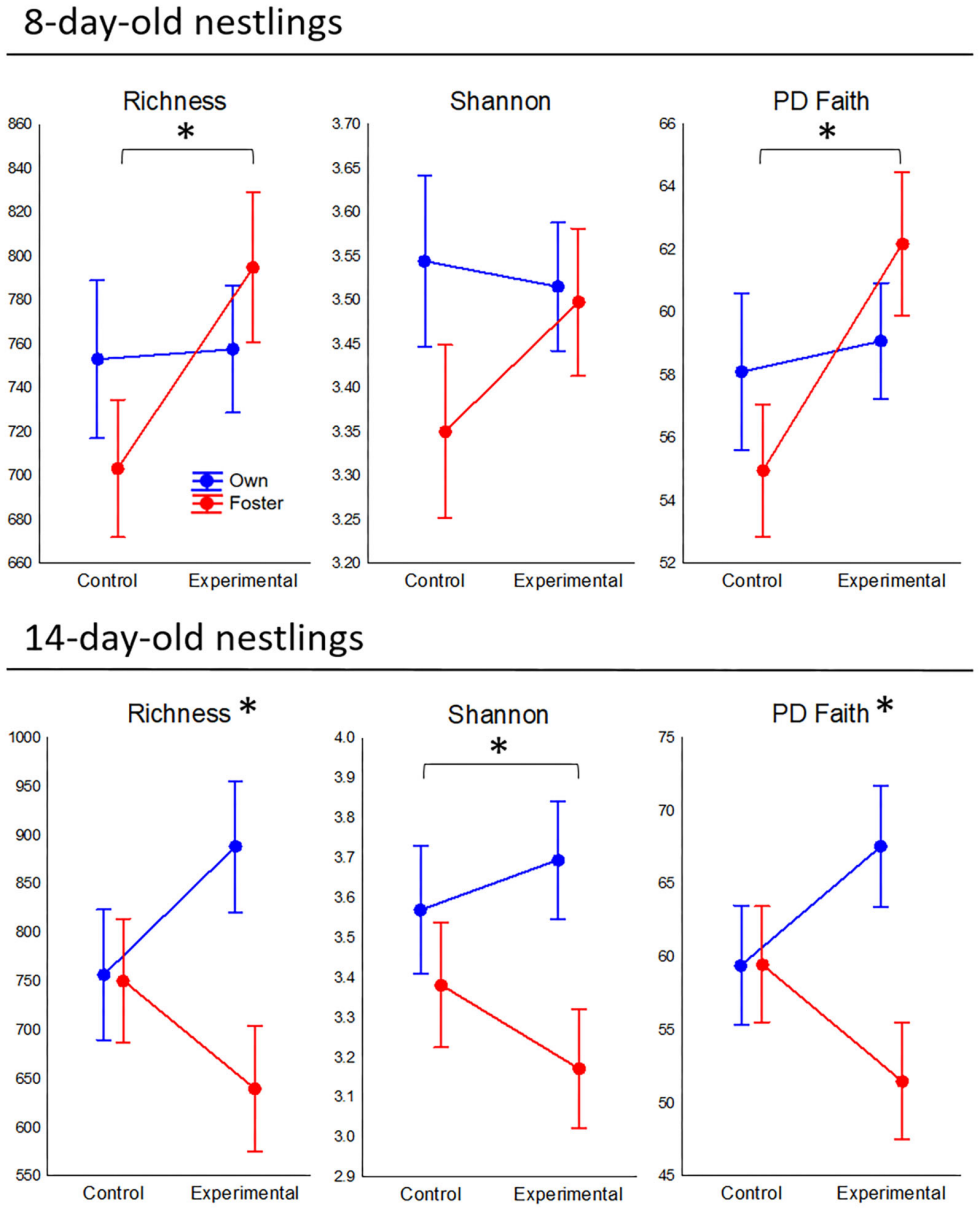
Weighted means (± SE) of alpha diversity indices (richness, Shannon, and PD Faith) for experimental and control starling nestlings from different origin at day 8 and day 14 of the nestling period (residuals after controlling for the effects of brood size and hatching date; see text). An asterisk next to the panel title indicates that the interaction term in the model was significant. Asterisks above brackets indicate significant post-hoc comparisons: * *P* < 0.05, ** *P* < 0.01, *** *P* < 0.001.

Experimentally increased parasitism significantly affected the alpha diversity of the gut microbiota of 14-day-old nestlings, but not that of 8-day-old nestlings (Table 2). The detected effects always appeared in interaction with sibling relatedness (alpha diversity values of samples from own nestlings tended to be higher in experimental nests, while the opposite tendencies were detected for foster nestlings (Fig. 3). In accordance, post-hoc comparisons showed that foster nestlings in experimental nests had higher values of richness (*P* = 0.018) and PD Faith diversities (*P* = 0.004) than those in control nests (Fig. 3). Moreover, when considering samples from 14-day-old nestlings in experimental nests, alpha diversity of gut microbiota of own nestlings was higher than in their foster siblings in terms of richness, Shannon and PD Faith diversity (LSD post-hoc comparisons, *P* < 0.05, Fig. 3), while nestlings raised in control nests exhibited similar alpha diversity indices regardless of their relatedness (Fig. 3). Regarding beta diversity, no effects were detected for any distance metric at either 8 or 14 days of age (Table 2, ESM Fig. S1).

**Table 2:** Results from linear mixed models and perMANOVAs testing the effects of experimental treatment and sibling relatedness (own vs. foster nestlings) on alpha diversity indices (richness, Shannon and PD Faith) and beta diversity matrices (Bray–Curtis, Weighted Unifrac and Unweighted Unifrac), for 8 and 14-day-old starling nestlings.

Alpha-diversity	Richness		Shannon		PD faith	
Predictors	F_1,81 // 1,71_	*P-value*	F_1,81 // 1,71_	*P-value*	F_1,81 // 1,71_	*P-value*
8-day-old model						
Intercept	0.01	0.92	0.00	0.96	0.04	0.84
Treatment	0.11	0.75	0.14	0.71	0.02	0.89
Sibling relatedness	0.55	0.46	4.23	**0.04**	0.59	0.44
Treatment * Sibling relatedness	1.32	0.25	0.37	0.54	1.16	0.28
Nest ID (treatment)	13.97	**<.001**	3.04	**<.001**	13.68	**<.001**
14-day-old model						
Intercept	0.16	0.69	0.15	0.70	0.25	0.62
Treatment	0.61	0.44	0.10	0.75	0.60	0.44
Sibling relatedness	0.04	0.84	1.14	0.29	0.01	0.91
Treatment * Sibling relatedness	5.25	**0.02**	0.05	0.82	4.77	**0.03**
Nest ID (treatment)	2.76	**<.001**	1.71	**0.001**	3.29	**<.001**
**Beta-diversity**	**Bray–Curtis**		**Weighted unifrac**		**Unweighted unifrac**	
**Predictors**	**F_1,81 // 1,71_**	***P-value***	**F_1,81 // 1,71_**	***P-value***	**F_1,81 // 1,71_**	***P-value***
8-day-old model						
Treatment	0.91	0.58	0.70	0.33	1.01	0.41
Sibling relatedness	0.79	0.77	0.72	0.18	1.03	0.35
Treatment * Sibling relatedness	0.62	0.62	0.83	0.59	0.95	0.63
Nest ID (treatment)	1.59	**<.001**	1.50	**<.001**	1.98	**<.001**
14-day-old model						
Treatment	0.97	0.47	0.88	0.55	0.71	0.81
Sibling relatedness	1.33	0.11	1.30	0.19	1.07	0.26
Treatment * Sibling relatedness	1.10	0.30	1.08	0.36	1.02	0.40
Nest ID (treatment)	1.89	**<.001**	1.74	**<.001**	2.28	**<.001**

Nest identity nested within experimental treatment was included as a random factor. Interaction effects were tested in separate models that also included the main effects. Values in bold indicate significance at α < 0.05. F-tests correspond to F1,81 for 8-day-old and F 1,71 for 14-day-old models.

**Figure 4:**
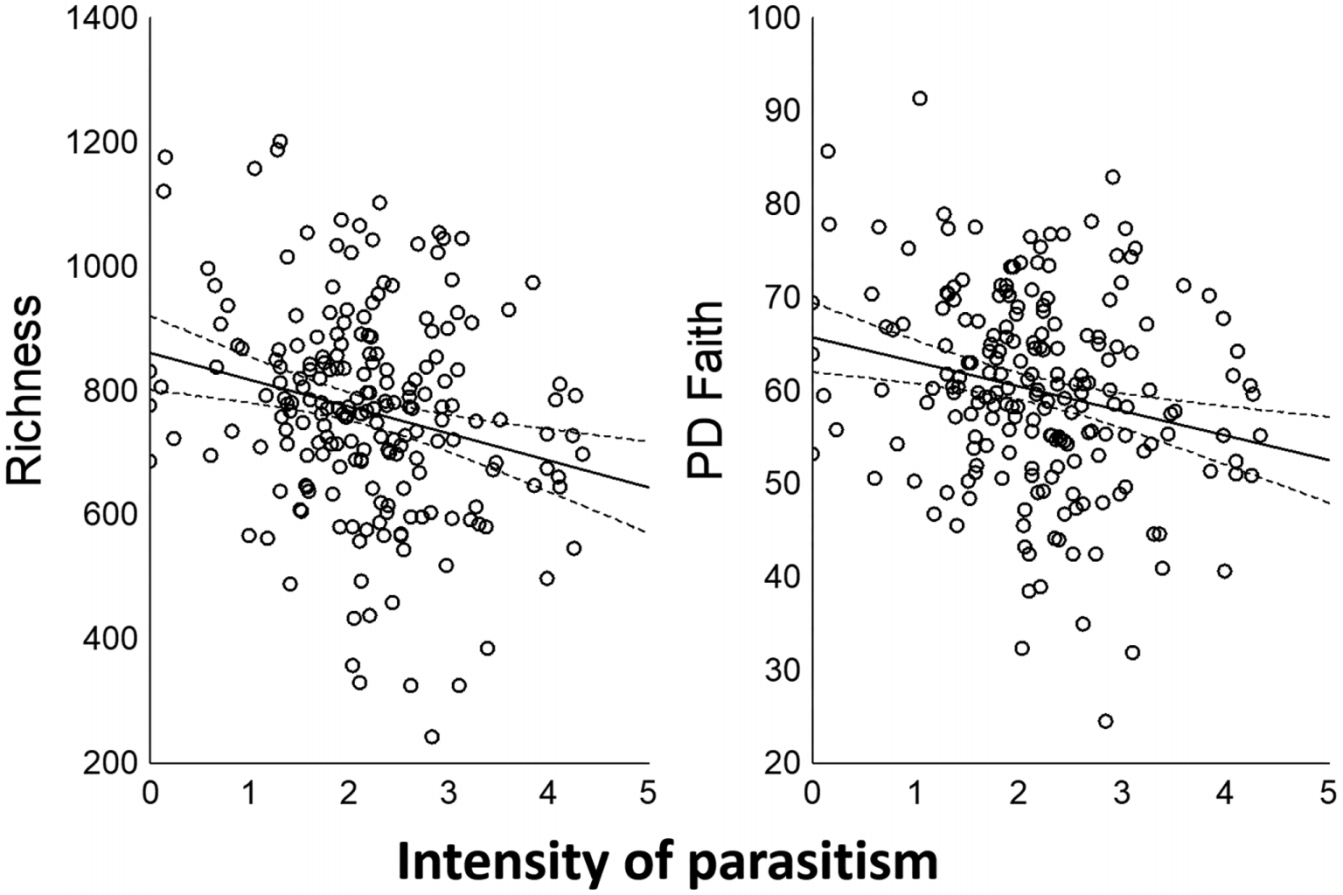
Regression plots showing the association between intensity of parasitism experienced by 14-day-old starling nestlings and alpha diversity indices (richness and PD Faith) (residuals after controlling for the effects of brood size and hatching date, see text).

**Figure 5:**
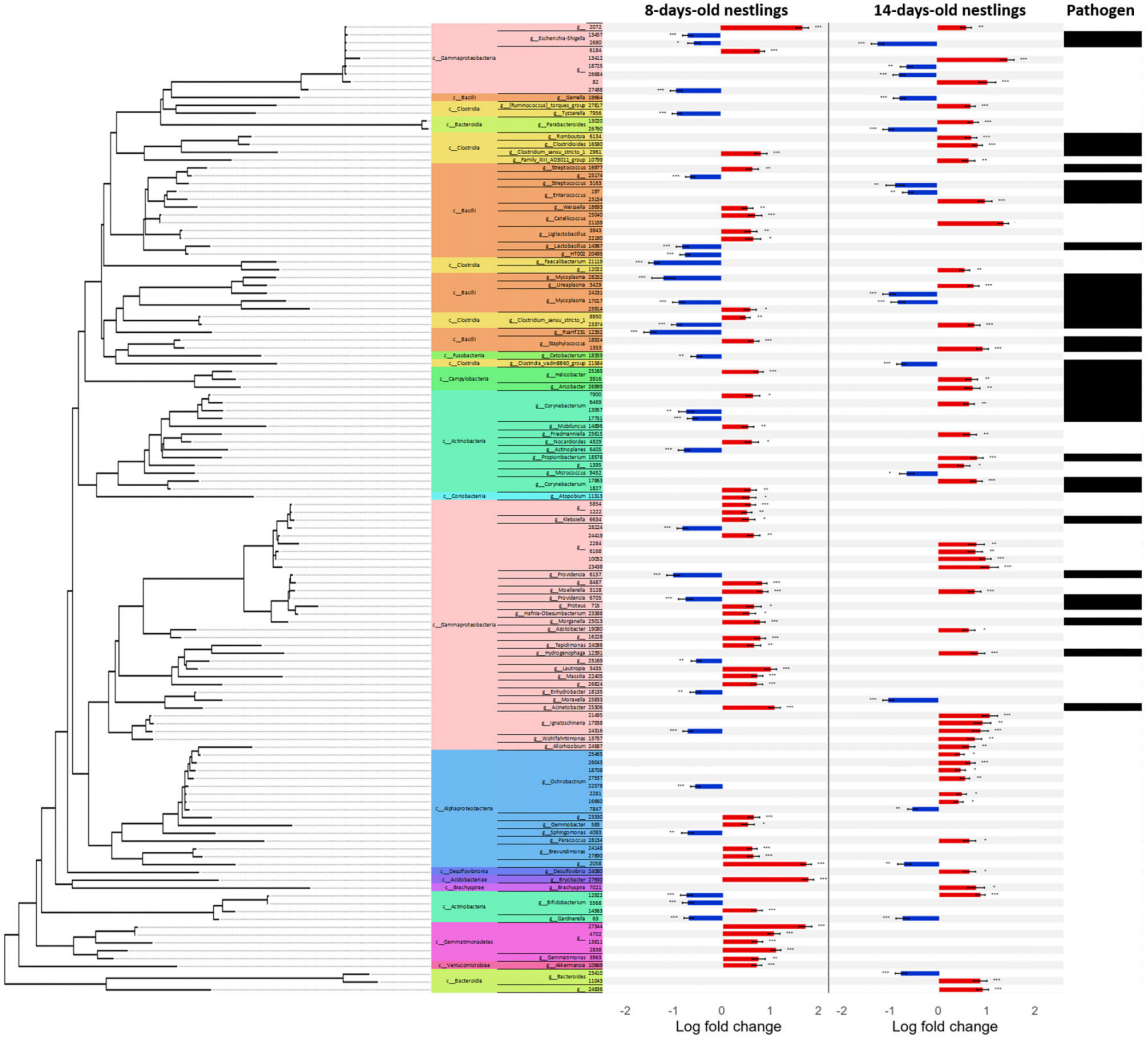
ANCOM-BC2 analysis of differential abundances expressed as log₂ fold-changes (LFC) for the top 123 ASVs for samples collected from 8 and 14-day-old starling nestlings. Bars pointing to the right (red) denote taxa whose abundance was significantly higher in nestlings from ectoparasite-supplemented boxes versus controls, while bars pointing to the left (blue) denote taxa whose abundance was significantly lower in the parasite treatment. Error bars represent the standard error of the estimated LFC. Statistical significance is annotated by asterisks: * *P* adj. ≤ 0.05; ** *P* adj. ≤ 0.01; *** *P* adj. ≤ 0.001. Each ASV is labelled by its class and genus. Following differential abundance testing, ASVs were screened for pathogenicity (see Materials and Methods).

Intensity of parasitism of 14, but not that of 8-day-old nestlings associated negatively with alpha diversity of their gut microbiota in terms of richness and PD Faith (ESM Table S4, Fig. 4). Similar effects were detected for beta diversity estimated as unweighted UniFrac distance matrix (ESM Table S4). Moreover, 123 ASVs significantly associated with treatment across all samples (ANCOM-BC2 analyses, adjusted *P* < 0.05; ESM Table S5), and, of these, 72 and 61 ASVs were biased to experimental treatment in 8 and 14-day-old nestlings, respectively (Fig. 5). Forty-three ASVs belonging to 27 genera were relatively more abundant in nestlings reared in control nests, while 90 ASVs belonging to 48 genera were more abundant in experimental nests (Fig. 5). Moreover, 26 of these genera include species catalogued as pathogenic in reference databases (NCBI Pathogen Detection, FAPROTAX) (Fig. 5).

### Microbial bacterial communities and nestling phenotypes

None of the estimated alpha-diversity indexes of gut bacterial communities of 8- and 14-day-old nestlings associated with their phenotypic characteristics (ESM Table S6, ESM Fig. S2). However, beta diversity estimates for bacterial communities of 8-day-old nestlings associated positively with carotenoid concentration and telomere dynamics when considering Bray–Curtis and weighted UniFrac distance matrices, respectively (Table 3). Moreover, beta diversity of the gut microbiota of 14-day-old nestlings associated positively with body mass, wing and tarsus length and carotenoid concentration of 14-day-old nestlings (Table 3). That was the case after controlling for the effect of hatching date, brood size and nestling relatedness.

**Table 3:**
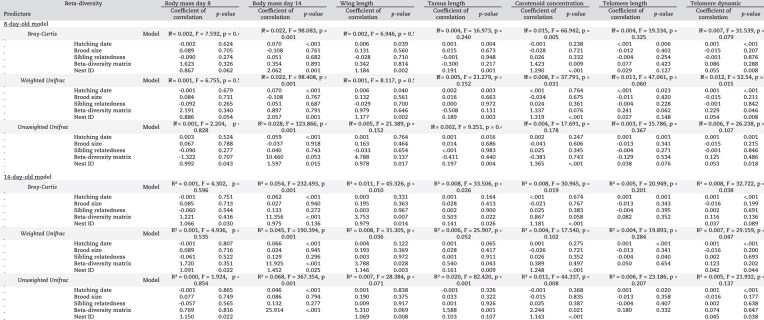
Results of MRMs exploring the correlation between beta diversity (Bray–Curtis, Weighted Unifrac, and Unweighted Unifrac) of gut bacterial community and phenotypic traits, for 8 and 14-day-old starling nestlings.

Euclidean distances on the nestling phenotypic traits were included as the dependent distance matrix, while distances on beta diversity were included as the first independent distance matrices. Binary matrices with information on nest ID, sibling relatedness and Euclidean distances on hatching date and brood size were also included to control for the effects of those factors. Number of permutations was set to 10 000. Values in bold indicate significance at α < 0.05.

**Figure 6:**
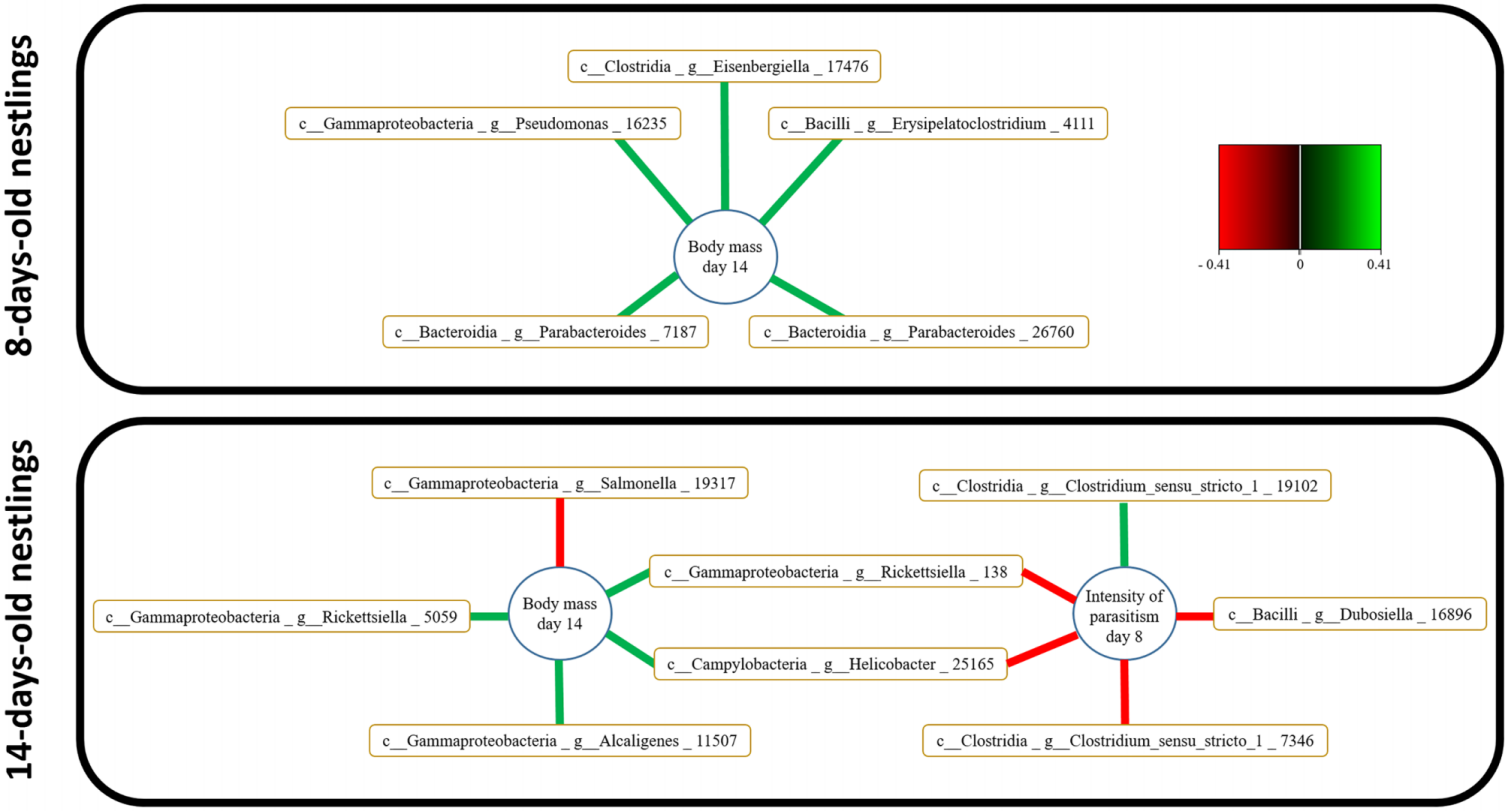
Bipartite network of sPLS associations between ASVs of the bacterial community of 8-day-old starling nestlings, and their body mass at day 14 (upper panel). The lower panel shows associations between ASVs of the bacterial community of 14-day-old nestlings and their body mass at the day of sampling and intensity of parasitism at intermediate nestling period. Rectangular nodes represent the five ASVs selected on each component 1; the central circular node is the dependent variable. Edges denote Pearson correlations |R| ≥ 0.25: green edges for positive correlations, red edges for negative correlations. Edge colour intensity scales with the absolute value of the correlation coefficient (lighter = stronger).

Finally, the first sPLS latent component of phenotypes and bacterial community composition of 8-day-old nestlings explained 12.07% of the variance in microbiota composition and 34.27% of the variance in phenotypic traits (ESM Appendix S1). It retained five ASVs (one positively and four negatively associated) and one phenotypic trait (body mass at day 14 as predictor) (Fig. 6). In 14-day-old nestlings, the two resulting latent components explained 7.58% and 8.88% of their variance that respectively associated with their body mass (variance explained = 34.49%) and parasitism intensity suffered 8 days after hatching (variance explained = 50.45%) (ESM Appendix S1). Each component retained five ASVs, one positively and four negatively related to body mass or to intensity of parasitism (Fig. 6). Two of those ASVs (g__Rickettsiella_138 and g__Helicobacter_25 165) appeared in both networks (Fig. 6).

## Discussion

Our main results posit that experimentally increased ectoparasitism by *Carnus hemapterus*, as well as ectoparasite intensity, influenced the diversity and composition of the gut microbiota of spotless starling nestlings. Some of these effects only appeared in experimental nestlings that were cross-fostered and therefore not genetically related to their social parents. Both experimental effects and correlative associations were significant for nestlings aged 14 days, i.e. at the end of the nestling period. Moreover, the experimental addition of ectoparasites to starling nests increased parasitism intensity and decreased body mass, but it was only detected at nestling age of day 8 (i.e. halfway through the nestling period). Experimentally increased parasitism also affected differential abundance of ASVs in the gut microbiota, including several potentially pathogenic genera, being more numerous in nestlings from nests with experimentally increased levels of parasitism. Finally, nestling phenotypic traits (body mass, wing and tarsus length, carotenoid concentration, and telomere dynamics) were associated with gut microbiota composition and/or with the relative abundance of specific bacterial genera. Taken together, these results suggest that the negative effects of ectoparasitism on nestling phenotype are, at least, partially mediated by the effect of ectoparasites on their gut microbiota, and that the genetic relatedness between nestlings and parents might contribute to counteract these negative effects. Below we discuss the importance and novelty of these results and the possible underlying mechanisms explaining the detected experimental and correlative associations.

Most evidence of the effect of parasites on gut microbiota of animals comes from mammals and experiments performed in laboratory conditions with intestinal parasites (e.g. Leung et al. 2018, Marsh et al. 2024). In birds, experimental disinfection of nests with permethrin (Ingala et al. 2021), or natural parasitism (Solomon et al. 2023) disrupted the gut microbiota of some of the study species. By directly adding parasites to experimental nests, our results add unequivocal evidence supporting the hypothesis that parasitism influences the gut microbiota of animals. The performed experiment with permethrin, however, affected the gut microbiota of eastern bluebirds (*Sialia sialis*) but not of tree swallows (*Tachycineta bicolor*) (Ingala et al. (2021), suggesting that the effects may depend on species-specific differences in strategies to counteract the negative effects of parasitism (i.e. antiparasitic defences including those of resistance and tolerance (Svensson and Råberg 2010, Soler and Soler 2017). Here we detected intraspecific differences in the hypothesized effects of experimentally increased parasitism associated with sibling relatedness (i.e. own vs foster nestlings). Interestingly, we detected such effects not only in the gut microbiota of nestlings, but also in the intensity of parasitism and body mass. In particular, the clearest effects were detected at intermediate age (day 8), with negative effects being specially detected in foreign nestlings, whereas at day 14 this pattern was not statistically supported and differences among treatment groups should therefore be interpreted cautiously. Infestation by *Carnus* commonly reaches its highest intensity at intermediate nestling ages (Dawson and Bortolotti 1997, Roulin 1998, Liker et al. 2001), which may explain why these effects were more apparent at day 8. Thus, in comparison with foster nestlings, those reared by their genetic parents better tolerated the experimental increase in ectoparasite infestation in their nests, particularly during this period of high parasite pressure.

The detected influence of genetic relatedness on the hypothesized effects of experimentally increased parasitism is unlikely explained by genetic or pre-hatching maternal effects directly related to antiparasitic defences because siblings of nestlings, which were considered as foster in one nest, were reared by their genetic parents in another nest. A more likely explanation is that, as it occurs in some species, parents were able to distinguish foster (i.e. parasitic) from own nestlings (Grim 2006, Attisano et al. 2018, Noh et al. 2018, 2021) and differentially invest more effort in the latter (Grim 2007, Grim 2017). Differential parental care toward own offspring has mainly been detected in terms of food allocation preferences ((Soler, M. 2017, Soler, M. et al. 2017). Yet, since adult birds increase nest sanitation effort in response to experimentally increased parasitism (Hurtrez‐Boussès et al. 2003, Mainwaring 2017), it is also possible that differential parental care is displayed also in behaviours aimed at cleaning the body of their offspring from ectoparasites. Diet is one of the main factors affecting microbiota (Wang et al. 2022, Huaiquipán et al. 2023), but it also influences performance (Davidson et al. 2020) and antiparasitic skills (Masello et al. 2018, Eleftherianos et al. 2024) of animals; traits that also associate with the gut microbiota (Davidson et al. 2021). Thus, whatever the parental care component involved, distinguishing own from foster siblings would allow adult starlings to invest differentially in the former and, then, would explain the detected effect of genetic relatedness of nestlings. Conspecific brood parasitism is relatively frequent in spotless starling nests (Monclús et al. 2017, Tomás et al. 2017, Azcárate-García et al. 2020), and defences in terms of parasitic egg recognition and ejection have apparently evolved in the species (Liénard et al. 2025). Thus, it is possible that recognizing own from stepsiblings had evolved in starlings, and that parents directed parental care to their genetic offspring differentially, which would partially counteract the hypothesized effect of experimentally increased parasite infestation on parasitism intensity and on gut microbiota. Future work focussed on parental behaviour in experimentally parasitized nests would clarify this possibility.

Independently of the mechanisms explaining the observed effect of growing up with genetically related parents, our experimental and correlative results strongly support the hypothesized link between parasitism and gut microbiota, whereby parasites may alter host condition, immune activity and the gut environment, ultimately affecting microbial diversity and composition (Broadhurst et al. 2012, Lee et al. 2020, Ingala et al. 2021, Guiver et al. 2022, Love et al. 2024, Wang et al. 2024). Both experimental effects on alpha diversity and the association with intensity of parasitism appeared in nestlings that were close to fledging age (day 14). This microbiome response delayed in time induced by a perturbation is a consistent pattern reported in other studies (van Veelen et al. 2020, Campos et al. 2025), so it is possible that early-life parasitism effects on gut microbiota become detectable only later as exposure accumulates and hosts develop. However, the relative abundance of several bacterial genera in the gut microbiota of 8 and 14-day-old nestlings was associated with the experimental treatment, suggesting that the effect of early exposure to parasitism still influences nestling microbiota both at intermediate age and close to fledging. As predicted, some of the bacterial genera that show higher abundance in experimental nests are opportunistic pathogens associated with skin [*Staphylococcus* (Hatlen and Miller 2021)], urogenital infections [*Ureaplasma (*Jhaveri and Lasalvia 2019)] and intestinal wounds (*Clostridioides, Helicobacter* and *Arcobacter* (Ramees et al. 2017, Fathima et al. 2022, Ali and AlHussaini 2024), which may suggest that it is an effect of wounds provoked by *Carnus* parasitism. In contrast, others genera are specifically more abundant in control nestlings. *Gemella* is a typical commensal of the upper digestive tract (Torres-Morales et al. 2023), and it has been described that nestlings reared under relatively undisturbed conditions show higher abundances of this bacterium (Alba et al. 2023). *Faecalibacterium, Lactobacillus*, and *Cetobacterium* are markers of gut health that preserve epithelial barrier function and favour fermentative metabolism (Yu et al. 2015, Zhang et al. 2021, Qi et al. 2023). These taxa are consistently present in normal gut microbiota rather than associated with dysbiosis typically produced by parasites (Wang et al. 2024), and were relatively more abundant in control nestlings. Nevertheless, because differential abundance analyses involve a large number of ASVs, these results should be interpreted cautiously and mainly as exploratory patterns that require further confirmation.

Our results also showed correlative links between composition of gut microbiota and both different aspects of nestling phenotype (i.e. body mass, carotenoid concentration and telomere dynamics) and parasitism intensity at intermediate and/or late nestling period. These associations could be explained because the gut microbiota can influence nutrient assimilation (Rowland et al. 2018), with potential consequences for body mass and circulating carotenoid levels. Alternatively, parasite-induced changes in the gut microbiota may contribute to physiological stress during development (Al-Rashidi and El-Wakil 2024), which could be associated with telomere shortening. Moreover, relative abundance of some bacterial taxa associated with body mass, a key predictor of survival and recruitment in bird populations (Moreno et al. 2005), while other bacterial were associated with parasite intensity. Together, these patterns suggest a tripartite association between parasitism, gut microbiota and nestling phenotypic conditions. For example, consistent with previous studies (Woting et al. 2014, Soares et al. 2024), *Erysipelatoclostridium* was positively associated with body mass, whereas Salmonella was negatively associated with body mass for close-to-fledge nestlings. In addition, *Dubosiella* and *Rickettsiella* were negatively associated with parasitism intensity at intermediate ages. *Dubosiella* has been linked to immune tolerance and maintenance of the mucosal barrier (Zhang et al. 2024), which could limit parasite impact. In contrast, *Rickettsiella* is a typical endosymbiont/entomopathogen of arthropods (Floriano et al. 2025). Strikingly, two bacterial strains that associated with body mass and intensity of parasitism were also differentially more abundant in nestlings reared in control or experimental nests. *Parabacteroides* was positively associated with body mass and relatively more abundant in control nests, being typically found in the gut microbiota of animals and known as specialist saccharolytic fermenters producing propionate and other SCFAs that enhance host growth (Zhang et al. 2023, Liu et al. 2025). Surprisingly, the relative abundance of a *Helicobacter* strain, a genus that include potentially pathogenic bacteria (Kusters Johannes et al. 2006, Javed et al. 2017) was positively associated with body mass and negatively associated with intensity of parasitism, but were also more abundant in experimental nests. Since the experimentally increased parasitism had negative effect on both nestlings’ body mass and intensity of parasitism, further investigations are necessary to clarify these apparent contradictory results.

## Conclusion

In conclusion, our experimental and correlative results support several predictions of the hypothesis suggesting that the well-known effects of parasitism on nestling growth is partially mediated by the changes in the microbiota that ectoparasites induce. Notably, these effects mainly appear in experimental foster nestlings suggesting that genetic relatedness between parents and offspring, or other parental effects, could buffer the negative effects of parasites on the gut microbiome, a possibility worth exploring in the near future.

## Supplementary Material

fiag079_Supplemental_File

## Data Availability

Raw paired-end 16S rRNA gene amplicon sequencing reads have been deposited in the European Nucleotide Archive under project accession: PRJEB113682 (https://www.ebi.ac.uk/ena/browser/view/PRJEB113682). The metadata (ASV and taxonomy tables, phylogenetic trees) and analysis code used in this paper have been deposited in the CSIC repository (https://digital.csic.es/handle/10261/436029). All other relevant data are available in the Supplementary materials.
