# Supplementary material for: Ectoparasitism and the gut microbiota: a cross-fostering experiment in a wild passerine
Source: FEMS Microbiol Ecol. 2026 Jul 17;102(8):fiag079. doi: 10.1093/femsec/fiag079 (PMC13403908; doi:10.1093/femsec/fiag079)
Supplement: fiag079_Supplemental_File [file fiag079_supplemental_file.docx]

**Supporting Information**

Ectoparasitism and the gut microbiota: a cross-fostering experiment in a wild passerine

Antonio José García-Núñez^1^*, Juan José Soler^1,2^, Jordi Figuerola^3,4^, Libya González-Dávalos^1^, José Antonio Morillo^1^, Cristina Ruiz-Castellano^1^, B. Irene Tieleman^5^, Manuel Martínez-Bueno^2,6^ and Gustavo Tomás^1,2^*

^1^Departamento de Ecología Funcional y Evolutiva, Estación Experimental de Zonas Áridas (CSIC), 04120 Almería, Spain

^2^Unidad Asociada (CSIC): Coevolución: cucos, hospedadores y bacterias simbiontes, Universidad de Granada, 18071, Granada, Spain

^3^Estación Biológica de Doñana (CSIC), 41092 Sevilla, Spain

^4^Centro de Investigación Biomedica en Red Epidemiología y Salud Pública (CIBERESP), 28029 Madrid, Spain

^5^Groningen Institute for Evolutionary Life Sciences (GELIFES), University of Groningen, Nijenborgh 7, 9747 AG Groningen, The Netherlands

^6^Departamento de Microbiología, Universidad de Granada, 18071, Granada, Spain ***Correspondence:**Corresponding authors

agarcia@eeza.csic.es, gtomas@eeza.csic.es

**ESM Appendix S1. Extended Details of Material and Methods.**

*Detecting specific bacterial taxa that associated with phenotype and intensity of parasitism*

To search for specific bacterial strains related to nestling phenotypic traits, we used sparse Partial Least Squares or Projection to Latent Structures (sPLS) models as implemented in “mixOmics” v6.22.0 package (Rohart *et al*., 2017) in R. For the sPLS implementation we used the regression mode, which fits a linear regression between high dimension datasets, assuming an asymmetric role of the dependent matrix (here phenotype) and the explanatory matrix (here microbial data). Only the first latent component for body mass at day 14 exceeded the established recommended threshold of Q2= 0.1 (Wold *et al*., 2001) for 8 (Q2= 0.103) and 14 (Q2= 0.11) days old nestlings, which was also identified as the optimal phenotypic variable during the tuning process. For the sPLS model, we used the Mfold cross-validation method with 50 repeats and 10 folds.

We also used an sPLS model to identify ASVs associated with intensity of parasitism, using the same settings as above (regression mode; Mfold cross-validation with 50 repeats and 10 folds). This analysis likewise yielded a single informative component for intensity of parasitism at day 8 with Q2 = 0.12 for 14 days old nestlings.

Rohart, F., Gautier, B., Singh, A. & Lê Cao, K.-A. 2017 mixOmics: An R package for ‘omics feature selection and multiple data integration. *PLoS computational biology* **13**, e1005752.

Wold, S., Sjöström, M. & Eriksson, L. 2001 PLS-regression: a basic tool of chemometrics. *Chemometrics and Intelligent Laboratory Systems* **58**, 109-130.

**ESM Figure S1**. Principal coordinate analyses on Bray-Curtis, weighted and unweighted UniFrac distances of microbiotas of control and experimental starling nestlings depending on sibling relatedness (own or foster). We show percentage of variance explained by the first two axes. Ellipses represent the 90% confidence interval of the points for each group.


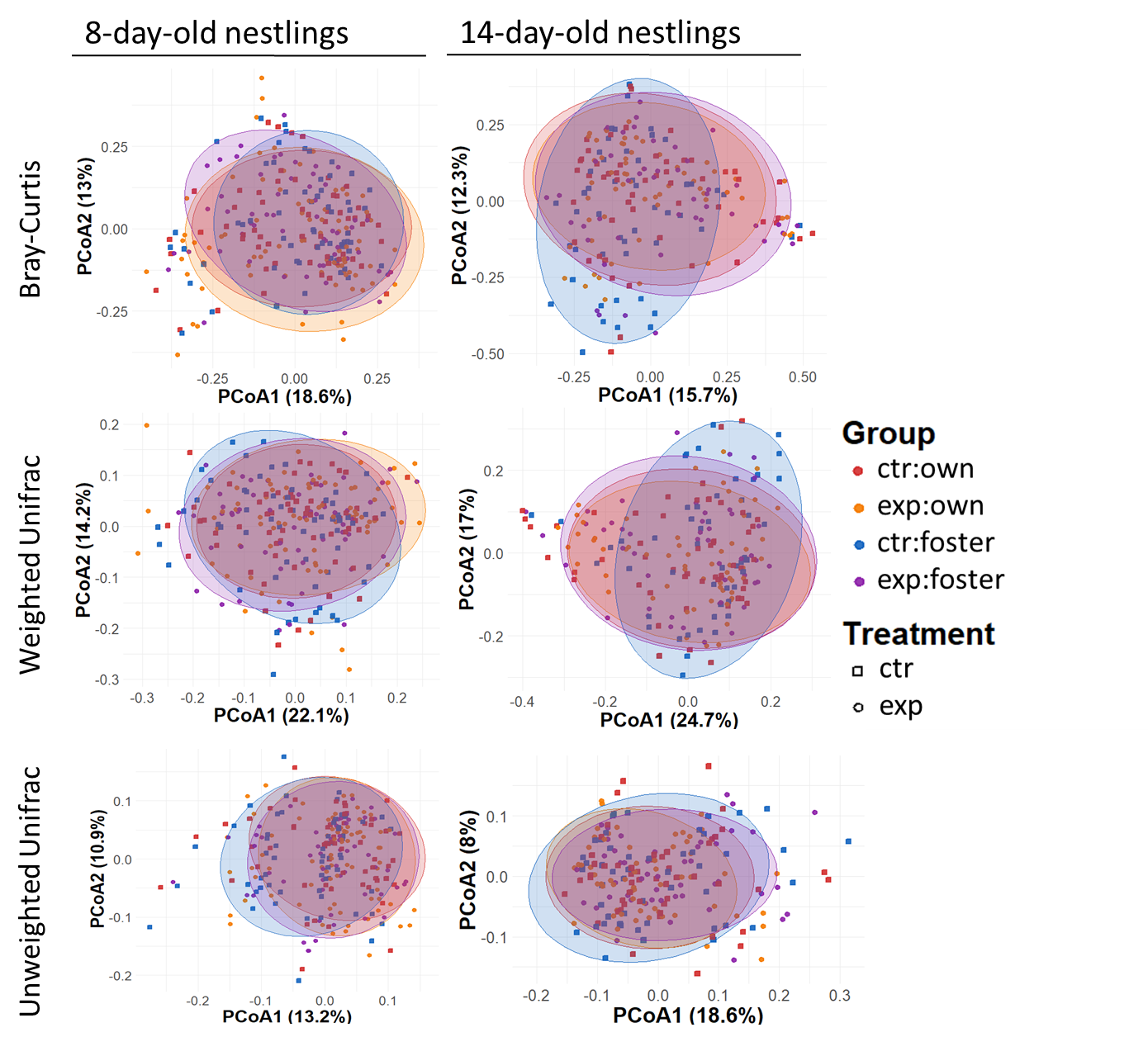


**ESM Figure S2**. Regression plots showing the association between alpha diversity indices and different phenotypic traits in starling nestlings (residuals after controlling for the effects of brood size and hatching date, see text). The Shannon index is related to tarsus length in 8-day-old nestlings, whereas richness and PD Faith are related to wing length in 14-day-old nestlings.


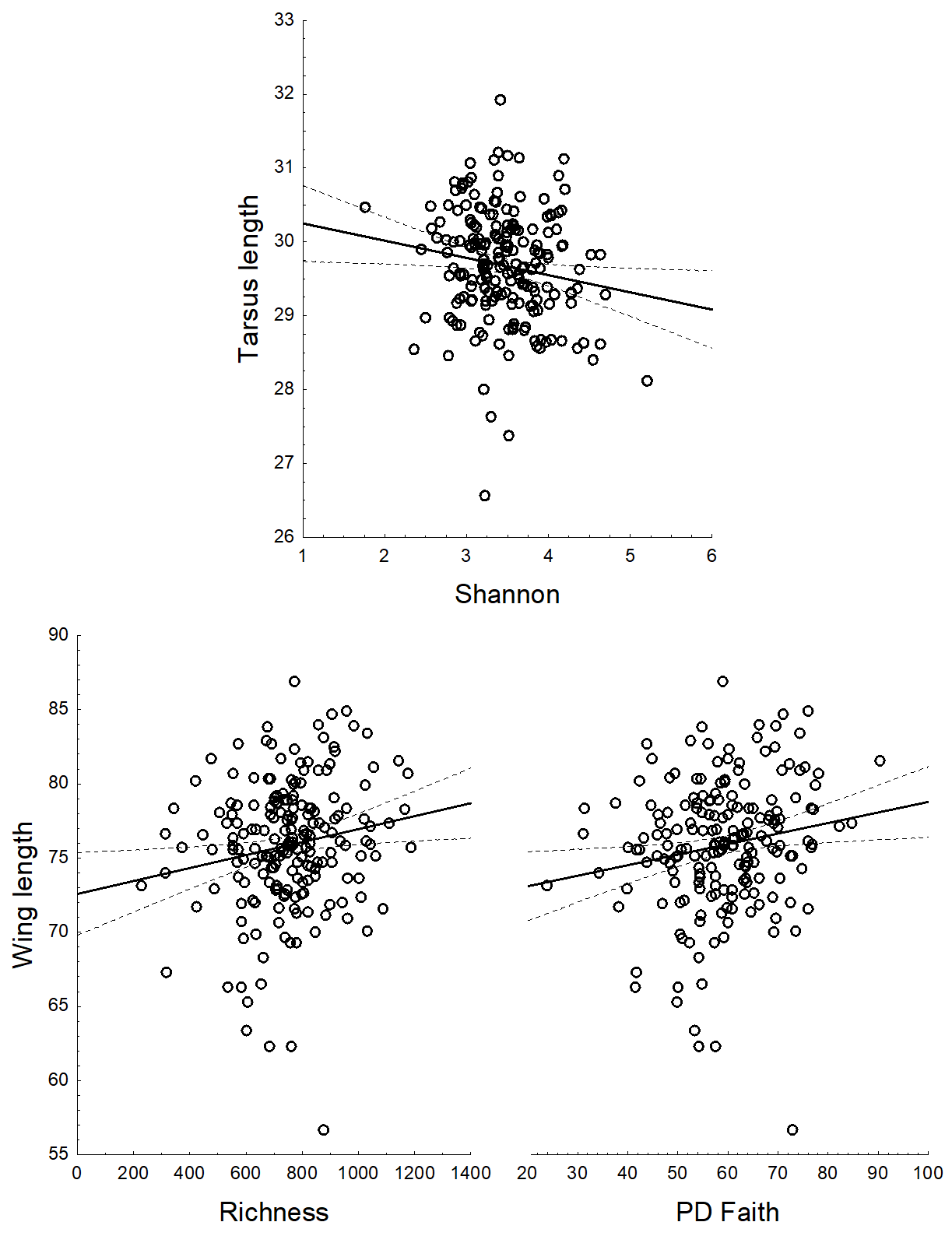


**Supporting tables**

**ESM Table S1**. Results from linear regression models testing the effects of hatching date and brood size on the intensity of parasitism, following a residualization approach, for 8 and 14-day-old starling nestlings. Values in bold indicate significance at α < 0.05.

|  |  |  |  | **8-day-old model** | | |  | **14-day-old model** | | |
| --- | --- | --- | --- | --- | --- | --- | --- | --- | --- | --- |
| **Dependent variable** | |  |  | **Adjusted R^2^** | **F** | ***p-value*** |  | **Adjusted R^2^** | **F** | ***p-value*** |
|  | Intensity of parasitism | |  | 0.085 | 12.90 | **<.001** |  | 0.046 | 6.15 | **<.001** |

**ESM Table S2**. Results from linear multiregression models and perMANOVAs testing the effects of hatching date and brood size on alpha diversity indices (Observed ASVs, Shannon and PD Faith) and beta diversity matrices (Bray-Curtis, Weighted Unifrac and Unweighted Unifrac), following a residualization approach, for 8 and 14-day-old starling nestlings. Values in bold indicate significance at α < 0.05.

|  |  |  |  | **8-day-old model** | | |  | **14-day-old model** | | |
| --- | --- | --- | --- | --- | --- | --- | --- | --- | --- | --- |
| **Dependent variables** | |  |  | **Adjusted R^2^** | **F_1,81_** | ***p-value*** |  | **Adjusted R^2^** | **F_1,71_** | ***p-value*** |
| Alpha diversity | |  |  |  |  |  |  |  |  |  |
|  | Richness | |  | 0.201 | 33.15 | **<.001** |  | 0.046 | 6.01 | **0.003** |
|  | Shannon |  |  | 0.071 | 10.76 | **<.001** |  | 0.016 | 2.72 | 0.07 |
|  | PD Faith |  |  | 0.211 | 35.04 | **<.001** |  | 0.059 | 7.55 | **<.001** |
|  |  |  |  |  |  |  |  |  |  |  |
| Beta diversity | |  |  |  |  |  |  |  |  |  |
|  | Bray–Curtis |  |  | 0.079 | 10.83 | **<.001** |  | 0.0540 | 5.91 | **<.001** |
|  | Weighted Unifrac | |  | 0.065 | 8.73 | **<.001** |  | 0.0479 | 5.20 | **<.001** |
|  | Unweighted Unifrac | |  | 0.091 | 12.61 | **<.001** |  | 0.0726 | 8.11 | **<.001** |

**ESM Table S3**. Results from linear multiregression models testing the effects of hatching date and brood size on nestling phenotypic traits (Observed ASVs, Shannon and PD Faith) and beta diversity matrices (Bray-Curtis, Weighted Unifrac and Unweighted Unifrac), following a residualization approach, for 8 and 14-day-old starling nestlings. Values in bold indicate significance at α < 0.05.

|  |  |  |  | **8-day-old model** | | |  | **14-day-old model** | | |
| --- | --- | --- | --- | --- | --- | --- | --- | --- | --- | --- |
| **Dependent variables** | |  |  | **Adjusted R^2^** | **F** | ***p-value*** |  | **Adjusted R^2^** | **F** | ***p-value*** |
|  |  |  |  |  |  |  |  |  |  |  |
|  | Body mass day 8 | |  | 0.046 | 6.01 | **0.003** |  | 0.044 | 5.75 | **0.004** |
|  | Body mass day 14 | |  | 0.221 | 30.38 | **<.001** |  | 0.223 | 30.60 | **<.001** |
|  | Wing length |  |  | 0.035 | 4.77 | **0.009** |  | 0.037 | 4.91 | **0.008** |
|  | Tarsus length |  |  | 0.036 | 4.89 | **0.008** |  | 0.038 | 5.02 | **0.007** |
|  | Carotenoid concentration | |  | 0.000 | 0.96 | 0.38 |  | 0.000 | 1.00 | 0.37 |
|  | Telomere length | |  | 0.000 | 1.03 | 0.36 |  | -0.001 | 0.87 | 0.42 |
|  | Telomere dynamic | |  | 0.009 | 1.96 | 0.14 |  | 0.009 | 1.89 | 0.15 |

**ESM Table S4**. Results from linear mixed models and perMANOVAs testing the association between intensity of parasitism experienced by nestlings on alpha diversity indices (richness, Shannon and PD Faith) and beta diversity matrices (Bray-Curtis, Weighted Unifrac and Unweighted Unifrac), for 8 and 14-day-old starling nestlings. Nest identity was included as a random factor. Separate models were used to test associations between parasitism intensity at day 8 and microbial communities at days 8 and 14, as well as between parasitism intensity at day 14 and microbial communities at day 14. Values in bold indicate significance at α < 0.05. F-tests correspond to F_1,81_ for 8-day-old and F _1,72_ for 14-day-old models.

|  | | | | **Richness** |  |  | **Shannon** |  |  | **PD Faith** |  |
| --- | --- | --- | --- | --- | --- | --- | --- | --- | --- | --- | --- |
| **Predictors** | |  |  | **F_1,82 // 1,72_** | ***p-value*** |  | **F_1,82 // 1,72_** | ***p-value*** |  | **F_1,82 // 1,72_** | ***p-value*** |
| 8-day-old model | |  |  |  |  |  |  |  |  |  |  |
|  | Intercept |  |  | 0.25 | 0.62 |  | 0.01 | 0.93 |  | 0.05 | 0.83 |
|  | Sibling relatedness | |  | 0.02 | 0.89 |  | 1.14 | 0.29 |  | 0.00 | 0.94 |
|  | Intensity of parasitism day 8 | | | 0.74 | 0.39 |  | 0.01 | 0.93 |  | 0.39 | 0.53 |
|  | Nest ID |  |  | 2.71 | **<.001** |  | 1.69 | **0.002** |  | 3.27 | **<.001** |
|  |  |  |  |  |  |  |  |  |  |  |  |
| 14-day-old model | |  |  |  |  |  |  |  |  |  |  |
|  | Intercept |  |  | 1.87 | 0.17 |  | 1.25 | 0.26 |  | 1.56 | 0.21 |
|  | Sibling relatedness | |  | 0.38 | 0.54 |  | 3.94 | 0.05 |  | 0.41 | 0.52 |
|  | Intensity of parasitism day 8 | | | 0.34 | 0.56 |  | 0.06 | 0.80 |  | 0.00 | 0.98 |
|  | Intensity of parasitism day 14 | | | 7.17 | **0.01** |  | 2.04 | 0.16 |  | 6.74 | **0.01** |
|  | Nest ID |  |  | 14.51 | **<.001** |  | 3.05 | **<.001** |  | 14.16 | **<.001** |
|  |  |  |  |  |  |  |  |  |  |  |  |
| **Beta-diversity** | | | | **Bray-Curtis** |  |  | **Weighted Unifrac** | |  | **Unweighted Unifrac** | |
| **Predictors** | |  |  | **F_1,82 // 1,72_** | ***p-value*** |  | **F_1,82 // 1,72_** | ***p-value*** |  | **F_1,82 // 1,72_** | ***p-value*** |
| 8-day-old model | |  |  |  |  |  |  |  |  |  |  |
|  | Sibling relatedness | |  | 1.04 | 0.36 |  | 1.42 | 0.14 |  | 1.43 | 0.10 |
|  | Intensity of parasitism day 8 | | | 0.62 | 0.92 |  | 0.42 | 0.97 |  | 0.72 | 0.81 |
|  | Nest ID |  |  |  |  |  |  |  |  |  |  |
|  |  |  |  |  |  |  |  |  |  |  |  |
| 14-day-old model | |  |  |  |  |  |  |  |  |  |  |
|  | Sibling relatedness | |  | 1.42 | 0.22 |  | 1.329 | 0.186 |  | 1.009 | 0.418 |
|  | Intensity of parasitism day 8 | | | 0.98 | 0.39 |  | 0.92 | 0.50 |  | 1.46 | 0.09 |
|  | Intensity of parasitism day 14 | | | 1.48 | 0.09 |  | 0.98 | 0.41 |  | 1.96 | **0.02** |
|  | Nest ID |  |  | 1.80 | 0.08 |  |  |  |  |  |  |

**ESM Table S5**. **List of differentially abundant 123 ASVs identified by ANCOM-BC2** associated with treatment for 8 and 14-day-old starling nestlings. For each ASV, the taxonomic assignment (Kingdom, Phylum, Class, Order, Family and Genus), the ASV number, and the full identifier used in the study (ASV ID) are provided.

| **Kingdom** | **Phylum** | **Class** | **Order** | **Family** | **Genus** | **ASV number** | **ASV ID** |
| --- | --- | --- | --- | --- | --- | --- | --- |
| k__Bacteria | p__Acidobacteriota | c__Acidobacteriae | o__Bryobacterales | f__Bryobacteraceae | g__Bryobacter | 27630 | c__Acidobacteriae _ g__Bryobacter _ 27630 |
| k__Bacteria | p__Actinobacteriota | c__Actinobacteria | o__Micrococcales | f__Micrococcaceae | g__ | 1395 | c__Actinobacteria _ g__ _ 1395 |
| k__Bacteria | p__Actinobacteriota | c__Actinobacteria | o__Micromonosporales | f__Micromonosporaceae | g__Actinoplanes | 6405 | c__Actinobacteria _ g__Actinoplanes _ 6405 |
| k__Bacteria | p__Actinobacteriota | c__Actinobacteria | o__Bifidobacteriales | f__Bifidobacteriaceae | g__Bifidobacterium | 12922 | c__Actinobacteria _ g__Bifidobacterium _ 12922 |
| k__Bacteria | p__Actinobacteriota | c__Actinobacteria | o__Bifidobacteriales | f__Bifidobacteriaceae | g__Bifidobacterium | 14563 | c__Actinobacteria _ g__Bifidobacterium _ 14563 |
| k__Bacteria | p__Actinobacteriota | c__Actinobacteria | o__Bifidobacteriales | f__Bifidobacteriaceae | g__Bifidobacterium | 5568 | c__Actinobacteria _ g__Bifidobacterium _ 5568 |
| k__Bacteria | p__Actinobacteriota | c__Actinobacteria | o__Corynebacteriales | f__Corynebacteriaceae | g__Corynebacterium | 13957 | c__Actinobacteria _ g__Corynebacterium _ 13957 |
| k__Bacteria | p__Actinobacteriota | c__Actinobacteria | o__Corynebacteriales | f__Corynebacteriaceae | g__Corynebacterium | 17751 | c__Actinobacteria _ g__Corynebacterium _ 17751 |
| k__Bacteria | p__Actinobacteriota | c__Actinobacteria | o__Corynebacteriales | f__Corynebacteriaceae | g__Corynebacterium | 17863 | c__Actinobacteria _ g__Corynebacterium _ 17863 |
| k__Bacteria | p__Actinobacteriota | c__Actinobacteria | o__Corynebacteriales | f__Corynebacteriaceae | g__Corynebacterium | 1827 | c__Actinobacteria _ g__Corynebacterium _ 1827 |
| k__Bacteria | p__Actinobacteriota | c__Actinobacteria | o__Corynebacteriales | f__Corynebacteriaceae | g__Corynebacterium | 6469 | c__Actinobacteria _ g__Corynebacterium _ 6469 |
| k__Bacteria | p__Actinobacteriota | c__Actinobacteria | o__Corynebacteriales | f__Corynebacteriaceae | g__Corynebacterium | 7900 | c__Actinobacteria _ g__Corynebacterium _ 7900 |
| k__Bacteria | p__Actinobacteriota | c__Actinobacteria | o__Propionibacteriales | f__Propionibacteriaceae | g__Friedmanniella | 25615 | c__Actinobacteria _ g__Friedmanniella _ 25615 |
| k__Bacteria | p__Actinobacteriota | c__Actinobacteria | o__Bifidobacteriales | f__Bifidobacteriaceae | g__Gardnerella | 63 | c__Actinobacteria _ g__Gardnerella _ 63 |
| k__Bacteria | p__Actinobacteriota | c__Actinobacteria | o__Micrococcales | f__Micrococcaceae | g__Micrococcus | 9452 | c__Actinobacteria _ g__Micrococcus _ 9452 |
| k__Bacteria | p__Actinobacteriota | c__Actinobacteria | o__Actinomycetales | f__Actinomycetaceae | g__Mobiluncus | 14896 | c__Actinobacteria _ g__Mobiluncus _ 14896 |
| k__Bacteria | p__Actinobacteriota | c__Actinobacteria | o__Propionibacteriales | f__Nocardioidaceae | g__Nocardioides | 4329 | c__Actinobacteria _ g__Nocardioides _ 4329 |
| k__Bacteria | p__Actinobacteriota | c__Actinobacteria | o__Propionibacteriales | f__Propionibacteriaceae | g__Propionibacterium | 18578 | c__Actinobacteria _ g__Propionibacterium _ 18578 |
| k__Bacteria | p__Proteobacteria | c__Alphaproteobacteria | o__ | f__ | g__ | 2058 | c__Alphaproteobacteria _ g__ _ 2058 |
| k__Bacteria | p__Proteobacteria | c__Alphaproteobacteria | o__Rhizobiales | f__Rhizobiaceae | g__ | 23330 | c__Alphaproteobacteria _ g__ _ 23330 |
| k__Bacteria | p__Proteobacteria | c__Alphaproteobacteria | o__Caulobacterales | f__Caulobacteraceae | g__Brevundimonas | 24148 | c__Alphaproteobacteria _ g__Brevundimonas _ 24148 |
| k__Bacteria | p__Proteobacteria | c__Alphaproteobacteria | o__Caulobacterales | f__Caulobacteraceae | g__Brevundimonas | 27890 | c__Alphaproteobacteria _ g__Brevundimonas _ 27890 |
| k__Bacteria | p__Proteobacteria | c__Alphaproteobacteria | o__Rhodobacterales | f__Rhodobacteraceae | g__Gemmobacter | 583 | c__Alphaproteobacteria _ g__Gemmobacter _ 583 |
| k__Bacteria | p__Proteobacteria | c__Alphaproteobacteria | o__Rhizobiales | f__Rhizobiaceae | g__Ochrobactrum | 16660 | c__Alphaproteobacteria _ g__Ochrobactrum _ 16660 |
| k__Bacteria | p__Proteobacteria | c__Alphaproteobacteria | o__Rhizobiales | f__Rhizobiaceae | g__Ochrobactrum | 18708 | c__Alphaproteobacteria _ g__Ochrobactrum _ 18708 |
| k__Bacteria | p__Proteobacteria | c__Alphaproteobacteria | o__Rhizobiales | f__Rhizobiaceae | g__Ochrobactrum | 22578 | c__Alphaproteobacteria _ g__Ochrobactrum _ 22578 |
| k__Bacteria | p__Proteobacteria | c__Alphaproteobacteria | o__Rhizobiales | f__Rhizobiaceae | g__Ochrobactrum | 2281 | c__Alphaproteobacteria _ g__Ochrobactrum _ 2281 |
| k__Bacteria | p__Proteobacteria | c__Alphaproteobacteria | o__Rhizobiales | f__Rhizobiaceae | g__Ochrobactrum | 25465 | c__Alphaproteobacteria _ g__Ochrobactrum _ 25465 |
| k__Bacteria | p__Proteobacteria | c__Alphaproteobacteria | o__Rhizobiales | f__Rhizobiaceae | g__Ochrobactrum | 26043 | c__Alphaproteobacteria _ g__Ochrobactrum _ 26043 |
| k__Bacteria | p__Proteobacteria | c__Alphaproteobacteria | o__Rhizobiales | f__Rhizobiaceae | g__Ochrobactrum | 27537 | c__Alphaproteobacteria _ g__Ochrobactrum _ 27537 |
| k__Bacteria | p__Proteobacteria | c__Alphaproteobacteria | o__Rhizobiales | f__Rhizobiaceae | g__Ochrobactrum | 7847 | c__Alphaproteobacteria _ g__Ochrobactrum _ 7847 |
| k__Bacteria | p__Proteobacteria | c__Alphaproteobacteria | o__Rhodobacterales | f__Rhodobacteraceae | g__Paracoccus | 28134 | c__Alphaproteobacteria _ g__Paracoccus _ 28134 |
| k__Bacteria | p__Proteobacteria | c__Alphaproteobacteria | o__Sphingomonadales | f__Sphingomonadaceae | g__Sphingomonas | 4083 | c__Alphaproteobacteria _ g__Sphingomonas _ 4083 |
| k__Bacteria | p__Firmicutes | c__Bacilli | o__Lactobacillales | f__ | g__ | 25174 | c__Bacilli _ g__ _ 25174 |
| k__Bacteria | p__Firmicutes | c__Bacilli | o__Lactobacillales | f__Catellicoccaceae | g__Catellicoccus | 21159 | c__Bacilli _ g__Catellicoccus _ 21159 |
| k__Bacteria | p__Firmicutes | c__Bacilli | o__Lactobacillales | f__Catellicoccaceae | g__Catellicoccus | 25040 | c__Bacilli _ g__Catellicoccus _ 25040 |
| k__Bacteria | p__Firmicutes | c__Bacilli | o__Lactobacillales | f__Enterococcaceae | g__Enterococcus | 197 | c__Bacilli _ g__Enterococcus _ 197 |
| k__Bacteria | p__Firmicutes | c__Bacilli | o__Lactobacillales | f__Enterococcaceae | g__Enterococcus | 23154 | c__Bacilli _ g__Enterococcus _ 23154 |
| k__Bacteria | p__Firmicutes | c__Bacilli | o__Staphylococcales | f__Gemellaceae | g__Gemella | 19664 | c__Bacilli _ g__Gemella _ 19664 |
| k__Bacteria | p__Firmicutes | c__Bacilli | o__Lactobacillales | f__Lactobacillaceae | g__HT002 | 20495 | c__Bacilli _ g__HT002 _ 20495 |
| k__Bacteria | p__Firmicutes | c__Bacilli | o__Lactobacillales | f__Lactobacillaceae | g__Lactobacillus | 14967 | c__Bacilli _ g__Lactobacillus _ 14967 |
| k__Bacteria | p__Firmicutes | c__Bacilli | o__Lactobacillales | f__Lactobacillaceae | g__Ligilactobacillus | 22180 | c__Bacilli _ g__Ligilactobacillus _ 22180 |
| k__Bacteria | p__Firmicutes | c__Bacilli | o__Lactobacillales | f__Lactobacillaceae | g__Ligilactobacillus | 3943 | c__Bacilli _ g__Ligilactobacillus _ 3943 |
| k__Bacteria | p__Firmicutes | c__Bacilli | o__Mycoplasmatales | f__Mycoplasmataceae | g__Mycoplasma | 17017 | c__Bacilli _ g__Mycoplasma _ 17017 |
| k__Bacteria | p__Firmicutes | c__Bacilli | o__Mycoplasmatales | f__Mycoplasmataceae | g__Mycoplasma | 24231 | c__Bacilli _ g__Mycoplasma _ 24231 |
| k__Bacteria | p__Firmicutes | c__Bacilli | o__Mycoplasmatales | f__Mycoplasmataceae | g__Mycoplasma | 25914 | c__Bacilli _ g__Mycoplasma _ 25914 |
| k__Bacteria | p__Firmicutes | c__Bacilli | o__Mycoplasmatales | f__Mycoplasmataceae | g__Mycoplasma | 28252 | c__Bacilli _ g__Mycoplasma _ 28252 |
| k__Bacteria | p__Firmicutes | c__Bacilli | o__RsaHf231 | f__RsaHf231 | g__RsaHf231 | 12352 | c__Bacilli _ g__RsaHf231 _ 12352 |
| k__Bacteria | p__Firmicutes | c__Bacilli | o__Staphylococcales | f__Staphylococcaceae | g__Staphylococcus | 1353 | c__Bacilli _ g__Staphylococcus _ 1353 |
| k__Bacteria | p__Firmicutes | c__Bacilli | o__Staphylococcales | f__Staphylococcaceae | g__Staphylococcus | 18924 | c__Bacilli _ g__Staphylococcus _ 18924 |
| k__Bacteria | p__Firmicutes | c__Bacilli | o__Lactobacillales | f__Streptococcaceae | g__Streptococcus | 16977 | c__Bacilli _ g__Streptococcus _ 16977 |
| k__Bacteria | p__Firmicutes | c__Bacilli | o__Lactobacillales | f__Streptococcaceae | g__Streptococcus | 3163 | c__Bacilli _ g__Streptococcus _ 3163 |
| k__Bacteria | p__Firmicutes | c__Bacilli | o__Mycoplasmatales | f__Mycoplasmataceae | g__Ureaplasma | 3429 | c__Bacilli _ g__Ureaplasma _ 3429 |
| k__Bacteria | p__Firmicutes | c__Bacilli | o__Lactobacillales | f__Lactobacillaceae | g__Weissella | 18693 | c__Bacilli _ g__Weissella _ 18693 |
| k__Bacteria | p__Bacteroidota | c__Bacteroidia | o__Bacteroidales | f__Prevotellaceae | g__ | 24836 | c__Bacteroidia _ g__ _ 24836 |
| k__Bacteria | p__Bacteroidota | c__Bacteroidia | o__Bacteroidales | f__Bacteroidaceae | g__Bacteroides | 11043 | c__Bacteroidia _ g__Bacteroides _ 11043 |
| k__Bacteria | p__Bacteroidota | c__Bacteroidia | o__Bacteroidales | f__Bacteroidaceae | g__Bacteroides | 23410 | c__Bacteroidia _ g__Bacteroides _ 23410 |
| k__Bacteria | p__Bacteroidota | c__Bacteroidia | o__Bacteroidales | f__Tannerellaceae | g__Parabacteroides | 13020 | c__Bacteroidia _ g__Parabacteroides _ 13020 |
| k__Bacteria | p__Bacteroidota | c__Bacteroidia | o__Bacteroidales | f__Tannerellaceae | g__Parabacteroides | 26760 | c__Bacteroidia _ g__Parabacteroides _ 26760 |
| k__Bacteria | p__Spirochaetota | c__Brachyspirae | o__Brachyspirales | f__Brachyspiraceae | g__Brachyspira | 7021 | c__Brachyspirae _ g__Brachyspira _ 7021 |
| k__Bacteria | p__Campylobacterota | c__Campylobacteria | o__Campylobacterales | f__Arcobacteraceae | g__Arcobacter | 26999 | c__Campylobacteria _ g__Arcobacter _ 26999 |
| k__Bacteria | p__Campylobacterota | c__Campylobacteria | o__Campylobacterales | f__Helicobacteraceae | g__Helicobacter | 25165 | c__Campylobacteria _ g__Helicobacter _ 25165 |
| k__Bacteria | p__Campylobacterota | c__Campylobacteria | o__Campylobacterales | f__Helicobacteraceae | g__Helicobacter | 3916 | c__Campylobacteria _ g__Helicobacter _ 3916 |
| k__Bacteria | p__Firmicutes | c__Clostridia | o__Oscillospirales | f__Ruminococcaceae | g__ | 12022 | c__Clostridia _ g__ _ 12022 |
| k__Bacteria | p__Firmicutes | c__Clostridia | o__Lachnospirales | f__Lachnospiraceae | g__[Ruminococcus]_torques_group | 27817 | c__Clostridia _ g__[Ruminococcus]_torques_group _ 27817 |
| k__Bacteria | p__Firmicutes | c__Clostridia | o__Clostridia_vadinBB60_group | f__Clostridia_vadinBB60_group | g__Clostridia_vadinBB60_group | 21584 | c__Clostridia _ g__Clostridia_vadinBB60_group _ 21584 |
| k__Bacteria | p__Firmicutes | c__Clostridia | o__Peptostreptococcales-Tissierellales | f__Peptostreptococcaceae | g__Clostridioides | 16580 | c__Clostridia _ g__Clostridioides _ 16580 |
| k__Bacteria | p__Firmicutes | c__Clostridia | o__Clostridiales | f__Clostridiaceae | g__Clostridium_sensu_stricto_1 | 23374 | c__Clostridia _ g__Clostridium_sensu_stricto_1 _ 23374 |
| k__Bacteria | p__Firmicutes | c__Clostridia | o__Clostridiales | f__Clostridiaceae | g__Clostridium_sensu_stricto_1 | 2961 | c__Clostridia _ g__Clostridium_sensu_stricto_1 _ 2961 |
| k__Bacteria | p__Firmicutes | c__Clostridia | o__Clostridiales | f__Clostridiaceae | g__Clostridium_sensu_stricto_1 | 8950 | c__Clostridia _ g__Clostridium_sensu_stricto_1 _ 8950 |
| k__Bacteria | p__Firmicutes | c__Clostridia | o__Oscillospirales | f__Ruminococcaceae | g__Faecalibacterium | 21119 | c__Clostridia _ g__Faecalibacterium _ 21119 |
| k__Bacteria | p__Firmicutes | c__Clostridia | o__Peptostreptococcales-Tissierellales | f__Anaerovoracaceae | g__Family_XIII_AD3011_group | 10799 | c__Clostridia _ g__Family_XIII_AD3011_group _ 10799 |
| k__Bacteria | p__Firmicutes | c__Clostridia | o__Peptostreptococcales-Tissierellales | f__Peptostreptococcaceae | g__Romboutsia | 6134 | c__Clostridia _ g__Romboutsia _ 6134 |
| k__Bacteria | p__Firmicutes | c__Clostridia | o__Lachnospirales | f__Lachnospiraceae | g__Tyzzerella | 7956 | c__Clostridia _ g__Tyzzerella _ 7956 |
| k__Bacteria | p__Actinobacteriota | c__Coriobacteriia | o__Coriobacteriales | f__Atopobiaceae | g__Atopobium | 11313 | c__Coriobacteriia _ g__Atopobium _ 11313 |
| k__Bacteria | p__Desulfobacterota | c__Desulfovibrionia | o__Desulfovibrionales | f__Desulfovibrionaceae | g__Desulfovibrio | 24080 | c__Desulfovibrionia _ g__Desulfovibrio _ 24080 |
| k__Bacteria | p__Fusobacteriota | c__Fusobacteriia | o__Fusobacteriales | f__Fusobacteriaceae | g__Cetobacterium | 18359 | c__Fusobacteriia _ g__Cetobacterium _ 18359 |
| k__Bacteria | p__Proteobacteria | c__Gammaproteobacteria | o__Enterobacterales | f__Enterobacteriaceae | g__ | 10052 | c__Gammaproteobacteria _ g__ _ 10052 |
| k__Bacteria | p__Proteobacteria | c__Gammaproteobacteria | o__Enterobacterales | f__Enterobacteriaceae | g__ | 1222 | c__Gammaproteobacteria _ g__ _ 1222 |
| k__Bacteria | p__Proteobacteria | c__Gammaproteobacteria | o__Enterobacterales | f__Enterobacteriaceae | g__ | 13412 | c__Gammaproteobacteria _ g__ _ 13412 |
| k__Bacteria | p__Proteobacteria | c__Gammaproteobacteria | o__Pseudomonadales | f__Pseudomonadaceae | g__ | 16228 | c__Gammaproteobacteria _ g__ _ 16228 |
| k__Bacteria | p__Proteobacteria | c__Gammaproteobacteria | o__Enterobacterales | f__Enterobacteriaceae | g__ | 18725 | c__Gammaproteobacteria _ g__ _ 18725 |
| k__Bacteria | p__Proteobacteria | c__Gammaproteobacteria | o__Enterobacterales | f__Enterobacteriaceae | g__ | 2072 | c__Gammaproteobacteria _ g__ _ 2072 |
| k__Bacteria | p__Proteobacteria | c__Gammaproteobacteria | o__Enterobacterales | f__Enterobacteriaceae | g__ | 2284 | c__Gammaproteobacteria _ g__ _ 2284 |
| k__Bacteria | p__Proteobacteria | c__Gammaproteobacteria | o__Enterobacterales | f__Enterobacteriaceae | g__ | 23438 | c__Gammaproteobacteria _ g__ _ 23438 |
| k__Bacteria | p__Proteobacteria | c__Gammaproteobacteria | o__Enterobacterales | f__Enterobacteriaceae | g__ | 24419 | c__Gammaproteobacteria _ g__ _ 24419 |
| k__Bacteria | p__Proteobacteria | c__Gammaproteobacteria | o__Burkholderiales | f__Alcaligenaceae | g__ | 25169 | c__Gammaproteobacteria _ g__ _ 25169 |
| k__Bacteria | p__Proteobacteria | c__Gammaproteobacteria | o__Enterobacterales | f__Enterobacteriaceae | g__ | 26684 | c__Gammaproteobacteria _ g__ _ 26684 |
| k__Bacteria | p__Proteobacteria | c__Gammaproteobacteria | o__Burkholderiales | f__Neisseriaceae | g__ | 26824 | c__Gammaproteobacteria _ g__ _ 26824 |
| k__Bacteria | p__Proteobacteria | c__Gammaproteobacteria | o__Enterobacterales | f__Enterobacteriaceae | g__ | 27488 | c__Gammaproteobacteria _ g__ _ 27488 |
| k__Bacteria | p__Proteobacteria | c__Gammaproteobacteria | o__Enterobacterales | f__Enterobacteriaceae | g__ | 28224 | c__Gammaproteobacteria _ g__ _ 28224 |
| k__Bacteria | p__Proteobacteria | c__Gammaproteobacteria | o__Enterobacterales | f__Enterobacteriaceae | g__ | 5854 | c__Gammaproteobacteria _ g__ _ 5854 |
| k__Bacteria | p__Proteobacteria | c__Gammaproteobacteria | o__Enterobacterales | f__Enterobacteriaceae | g__ | 6168 | c__Gammaproteobacteria _ g__ _ 6168 |
| k__Bacteria | p__Proteobacteria | c__Gammaproteobacteria | o__Enterobacterales | f__Enterobacteriaceae | g__ | 6184 | c__Gammaproteobacteria _ g__ _ 6184 |
| k__Bacteria | p__Proteobacteria | c__Gammaproteobacteria | o__Enterobacterales | f__Enterobacteriaceae | g__ | 82 | c__Gammaproteobacteria _ g__ _ 82 |
| k__Bacteria | p__Proteobacteria | c__Gammaproteobacteria | o__Enterobacterales | f__Morganellaceae | g__ | 8487 | c__Gammaproteobacteria _ g__ _ 8487 |
| k__Bacteria | p__Proteobacteria | c__Gammaproteobacteria | o__Pseudomonadales | f__Moraxellaceae | g__Acinetobacter | 25306 | c__Gammaproteobacteria _ g__Acinetobacter _ 25306 |
| k__Bacteria | p__Proteobacteria | c__Gammaproteobacteria | o__Pseudomonadales | f__Pseudomonadaceae | g__Azotobacter | 19080 | c__Gammaproteobacteria _ g__Azotobacter _ 19080 |
| k__Bacteria | p__Proteobacteria | c__Gammaproteobacteria | o__Pseudomonadales | f__Moraxellaceae | g__Enhydrobacter | 18135 | c__Gammaproteobacteria _ g__Enhydrobacter _ 18135 |
| k__Bacteria | p__Proteobacteria | c__Gammaproteobacteria | o__Enterobacterales | f__Enterobacteriaceae | g__Escherichia-Shigella | 13457 | c__Gammaproteobacteria _ g__Escherichia-Shigella _ 13457 |
| k__Bacteria | p__Proteobacteria | c__Gammaproteobacteria | o__Enterobacterales | f__Enterobacteriaceae | g__Escherichia-Shigella | 2680 | c__Gammaproteobacteria _ g__Escherichia-Shigella _ 2680 |
| k__Bacteria | p__Proteobacteria | c__Gammaproteobacteria | o__Enterobacterales | f__Hafniaceae | g__Hafnia-Obesumbacterium | 23388 | c__Gammaproteobacteria _ g__Hafnia-Obesumbacterium _ 23388 |
| k__Bacteria | p__Proteobacteria | c__Gammaproteobacteria | o__Burkholderiales | f__Comamonadaceae | g__Hydrogenophaga | 12391 | c__Gammaproteobacteria _ g__Hydrogenophaga _ 12391 |
| k__Bacteria | p__Proteobacteria | c__Gammaproteobacteria | o__Cardiobacteriales | f__Wohlfahrtiimonadaceae | g__Ignatzschineria | 17938 | c__Gammaproteobacteria _ g__Ignatzschineria _ 17938 |
| k__Bacteria | p__Proteobacteria | c__Gammaproteobacteria | o__Cardiobacteriales | f__Wohlfahrtiimonadaceae | g__Ignatzschineria | 21495 | c__Gammaproteobacteria _ g__Ignatzschineria _ 21495 |
| k__Bacteria | p__Proteobacteria | c__Gammaproteobacteria | o__Cardiobacteriales | f__Wohlfahrtiimonadaceae | g__Ignatzschineria | 24316 | c__Gammaproteobacteria _ g__Ignatzschineria _ 24316 |
| k__Bacteria | p__Proteobacteria | c__Gammaproteobacteria | o__Enterobacterales | f__Enterobacteriaceae | g__Klebsiella | 6634 | c__Gammaproteobacteria _ g__Klebsiella _ 6634 |
| k__Bacteria | p__Proteobacteria | c__Gammaproteobacteria | o__Burkholderiales | f__Burkholderiaceae | g__Lautropia | 3435 | c__Gammaproteobacteria _ g__Lautropia _ 3435 |
| k__Bacteria | p__Proteobacteria | c__Gammaproteobacteria | o__Burkholderiales | f__Oxalobacteraceae | g__Massilia | 22405 | c__Gammaproteobacteria _ g__Massilia _ 22405 |
| k__Bacteria | p__Proteobacteria | c__Gammaproteobacteria | o__Enterobacterales | f__Morganellaceae | g__Moellerella | 3128 | c__Gammaproteobacteria _ g__Moellerella _ 3128 |
| k__Bacteria | p__Proteobacteria | c__Gammaproteobacteria | o__Pseudomonadales | f__Moraxellaceae | g__Moraxella | 25833 | c__Gammaproteobacteria _ g__Moraxella _ 25833 |
| k__Bacteria | p__Proteobacteria | c__Gammaproteobacteria | o__Enterobacterales | f__Morganellaceae | g__Morganella | 25013 | c__Gammaproteobacteria _ g__Morganella _ 25013 |
| k__Bacteria | p__Proteobacteria | c__Gammaproteobacteria | o__Enterobacterales | f__Morganellaceae | g__Proteus | 715 | c__Gammaproteobacteria _ g__Proteus _ 715 |
| k__Bacteria | p__Proteobacteria | c__Gammaproteobacteria | o__Enterobacterales | f__Morganellaceae | g__Providencia | 6157 | c__Gammaproteobacteria _ g__Providencia _ 6157 |
| k__Bacteria | p__Proteobacteria | c__Gammaproteobacteria | o__Enterobacterales | f__Morganellaceae | g__Providencia | 6705 | c__Gammaproteobacteria _ g__Providencia _ 6705 |
| k__Bacteria | p__Proteobacteria | c__Gammaproteobacteria | o__Burkholderiales | f__Comamonadaceae | g__Tepidimonas | 24086 | c__Gammaproteobacteria _ g__Tepidimonas _ 24086 |
| k__Bacteria | p__Proteobacteria | c__Gammaproteobacteria | o__Cardiobacteriales | f__Wohlfahrtiimonadaceae | g__Wohlfahrtiimonas | 13757 | c__Gammaproteobacteria _ g__Wohlfahrtiimonas _ 13757 |
| k__Bacteria | p__Gemmatimonadota | c__Gemmatimonadetes | o__Gemmatimonadales | f__Gemmatimonadaceae | g__ | 13811 | c__Gemmatimonadetes _ g__ _ 13811 |
| k__Bacteria | p__Gemmatimonadota | c__Gemmatimonadetes | o__Gemmatimonadales | f__Gemmatimonadaceae | g__ | 27344 | c__Gemmatimonadetes _ g__ _ 27344 |
| k__Bacteria | p__Gemmatimonadota | c__Gemmatimonadetes | o__Gemmatimonadales | f__Gemmatimonadaceae | g__ | 2838 | c__Gemmatimonadetes _ g__ _ 2838 |
| k__Bacteria | p__Gemmatimonadota | c__Gemmatimonadetes | o__Gemmatimonadales | f__Gemmatimonadaceae | g__ | 4702 | c__Gemmatimonadetes _ g__ _ 4702 |
| k__Bacteria | p__Gemmatimonadota | c__Gemmatimonadetes | o__Gemmatimonadales | f__Gemmatimonadaceae | g__Gemmatimonas | 3963 | c__Gemmatimonadetes _ g__Gemmatimonas _ 3963 |
| k__Bacteria | p__Verrucomicrobiota | c__Verrucomicrobiae | o__Verrucomicrobiales | f__Akkermansiaceae | g__Akkermansia | 10669 | c__Verrucomicrobiae _ g__Akkermansia _ 10669 |

**ESM Table S6**. Results from linear mixed models testing the association between alpha diversity indices (richness, Shannon and PD Faith) on nestling phenotypic traits, for 8 and 14-day-old starling nestlings. Nest identity was included as a random factor. Values in bold indicate significance at α < 0.05.

| **Alpha-diversity** | | | | **Body mass**  **day 8** | |  | **Body mass**  **day 14** | |  | **Wing length** | |  | **Tarsus length** | |  | **Carotenoid concentration** | |  | **Telomere length** | |  | **Telomere dynamic** | |
| --- | --- | --- | --- | --- | --- | --- | --- | --- | --- | --- | --- | --- | --- | --- | --- | --- | --- | --- | --- | --- | --- | --- | --- |
| **Predictors** | |  |  | **F_1,72_** | ***p-value*** |  | **F_1,72_** | ***p-value*** |  | **F_1,72_** | ***p-value*** |  | **F_1,72_** | ***p-value*** |  | **F_1,72_** | ***p-value*** |  | **F_1,72_** | ***p-value*** |  | **F_1,72_** | ***p-value*** |
| 8-day-old model | | |  |  |  |  |  |  |  |  |  |  |  |  |  |  |  |  |  |  |  |  |  |
|  | *Richness* |  | Model | Adj. R^2^ = 0.109 | |  | Adj. R^2^ = 0.285 | |  | Adj. R^2^ = 0.294 | |  | Adj. R^2^ = 0.285 | |  | Adj. R^2^ = 0.661 | |  | Adj. R^2^ = 0.065 | |  | Adj. R^2^ = 0.152 | |
|  |  |  |  | 1.341 | 0.071 |  | 2.105 | <.001 |  | 2.159 | <.001 |  | 2.109 | <.001 |  | 6.424 | <.001 |  | 1.194 | 0.187 |  | 1.498 | 0.022 |
|  |  | Intercept |  | 0.037 | 0.847 |  | 0.000 | 1.000 |  | 0.156 | 0.693 |  | 0.037 | 0.847 |  | 0.484 | 0.488 |  | 0.131 | 0.718 |  | 0.023 | 0.880 |
|  |  | Sibling relatedness | | 0.013 | 0.908 |  | 0.914 | 0.341 |  | 1.948 | 0.165 |  | 0.061 | 0.805 |  | 1.824 | 0.179 |  | 0.585 | 0.446 |  | 1.999 | 0.160 |
|  |  | Alpha-diversity index | | 0.027 | 0.870 |  | 0.010 | 0.922 |  | 0.282 | 0.596 |  | 0.011 | 0.915 |  | 0.770 | 0.382 |  | 0.119 | 0.731 |  | 0.029 | 0.866 |
|  |  | Nest ID |  | 1.370 | 0.060 |  | 2.129 | **<.001** |  | 2.193 | **<.001** |  | 2.166 | **<.001** |  | 6.367 | **<.001** |  | 1.177 | 0.209 |  | 1.490 | **<.001** |
|  | *Shannon* |  | Model | Adj. R^2^ = 0.123 | |  | Adj. R^2^ = 0.288 | |  | Adj. R^2^ = 0.303 | |  | Adj. R^2^ = 0.303 | |  | Adj. R^2^ = 0.660 | |  | Adj. R^2^ = 0.066 | |  | Adj. R^2^ = 0.156 | |
|  |  |  |  | 1.390 | 0.050 |  | 2.121 | <.001 |  | 2.209 | <.001 |  | 2.205 | <.001 |  | 6.382 | <.001 |  | 1.197 | 0.184 |  | 1.514 | 0.019 |
|  |  | Intercept |  | 2.073 | 0.152 |  | 0.611 | 0.436 |  | 1.659 | 0.200 |  | 3.313 | 0.071 |  | 0.055 | 0.815 |  | 0.235 | 0.629 |  | 0.615 | 0.434 |
|  |  | Sibling relatedness | | 0.099 | 0.753 |  | 1.107 | 0.295 |  | 2.501 | 0.116 |  | 0.248 | 0.620 |  | 1.877 | 0.173 |  | 0.692 | 0.407 |  | 1.670 | 0.199 |
|  |  | Alpha-diversity index | | 2.070 | 0.153 |  | 0.543 | 0.463 |  | 1.954 | 0.164 |  | 3.239 | 0.074 |  | 0.094 | 0.760 |  | 0.260 | 0.611 |  | 0.650 | 0.421 |
|  |  | Nest ID |  | 1.412 | **0.044** |  | 2.144 | **<.001** |  | 2.243 | **<.001** |  | 2.261 | **<.001** |  | 6.318 | **<.001** |  | 1.224 | 0.158 |  | 1.521 | **0.019** |
|  | *PD Faith* |  | Model | Adj. R^2^ = 0.109 | |  | Adj. R^2^ = 0.288 | |  | Adj. R^2^ = 0.293 | |  | Adj. R^2^ = 0.285 | |  | Adj. R^2^ = 0.660 | |  | Adj. R^2^ = 0.064 | |  | Adj. R^2^ = 0.152 | |
|  |  |  |  | 1.342 | 0.071 |  | 2.123 | <.001 |  | 2.150 | <.001 |  | 2.109 | <.001 |  | 6.383 | <.001 |  | 1.191 | 0.190 |  | 1.499 | 0.021 |
|  |  | Intercept |  | 0.040 | 0.841 |  | 0.476 | 0.491 |  | 0.013 | 0.909 |  | 0.000 | 0.996 |  | 0.059 | 0.808 |  | 0.003 | 0.956 |  | 0.041 | 0.840 |
|  |  | Sibling relatedness | | 0.013 | 0.911 |  | 0.903 | 0.344 |  | 1.931 | 0.167 |  | 0.060 | 0.807 |  | 1.806 | 0.181 |  | 0.588 | 0.444 |  | 1.996 | 0.160 |
|  |  | Alpha-diversity index | | 0.055 | 0.815 |  | 0.616 | 0.434 |  | 0.001 | 0.976 |  | 0.006 | 0.936 |  | 0.109 | 0.741 |  | 0.001 | 0.972 |  | 0.047 | 0.830 |
|  |  | Nest ID |  | 1.376 | 0.057 |  | 2.136 | **<.001** |  | 2.187 | **<.001** |  | 2.166 | **<.001** |  | 6.367 | **<.001** |  | 1.176 | 0.210 |  | 1.467 | **0.029** |
|  |  |  |  |  |  |  |  |  |  |  |  |  |  |  |  |  |  |  |  |  |  |  |  |
| 14-day-old model | | |  |  |  |  |  |  |  |  |  |  |  |  |  |  |  |  |  |  |  |  |  |
|  | *Richness* |  | Model | Adj. R^2^ = 0.159 | |  | Adj. R^2^ = 0.318 | |  | Adj. R^2^ = 0.364 | |  | Adj. R^2^ = 0.337 | |  | Adj. R^2^ = 0.641 | |  | Adj. R^2^ = - 0.004 | |  | Adj. R^2^ = 0.124 | |
|  |  |  |  | 1.514 | 0.020 |  | 2.266 | <.001 |  | 2.555 | <.001 |  | 2.382 | <.001 |  | 5.862 | <.001 |  | 0.513 | 0.599 |  | 1.386 | 0.053 |
|  |  | Intercept |  | 1.277 | 0.260 |  | 0.181 | 0.671 |  | 3.635 | 0.058 |  | 0.153 | 0.696 |  | 0.002 | 0.967 |  | 0.895 | 0.345 |  | 0.135 | 0.714 |
|  |  | Sibling relatedness | | 0.027 | 0.871 |  | 1.254 | 0.265 |  | 2.163 | 0.144 |  | 0.178 | 0.674 |  | 2.476 | 0.118 |  | 0.130 | 0.718 |  | 2.776 | 0.098 |
|  |  | Alpha-diversity index | | 1.358 | 0.246 |  | 0.359 | 0.550 |  | 3.762 | 0.055 |  | 0.070 | 0.792 |  | 0.001 | 0.976 |  | 0.962 | 0.328 |  | 0.079 | 0.780 |
|  |  | Nest ID |  | 1.550 | **0.016** |  | 2.034 | **<.001** |  | 2.475 | **<.001** |  | 2.397 | **<.001** |  | 5.455 | **<.001** |  |  |  |  | 1.415 | **0.045** |
|  | *Shannon* |  | Model | Adj. R^2^ = 0.150 | |  | Adj. R^2^ = 0.319 | |  | Adj. R^2^ = 0.351 | |  | Adj. R^2^ = 0.337 | |  | Adj. R^2^ = 0.645 | |  | Adj. R^2^ = - 0.006 | |  | Adj. R^2^ = 0.124 | |
|  |  |  |  | 1.479 | 0.026 |  | 2.273 | <.001 |  | 2.472 | <.001 |  | 2.381 | <.001 |  | 5.941 | <.001 |  | 0.392 | 0.676 |  | 1.386 | 0.053 |
|  |  | Intercept |  | 0.003 | 0.956 |  | 0.342 | 0.560 |  | 1.268 | 0.262 |  | 0.001 | 0.978 |  | 0.955 | 0.330 |  | 0.768 | 0.382 |  | 0.116 | 0.734 |
|  |  | Sibling relatedness | | 0.058 | 0.810 |  | 1.031 | 0.312 |  | 1.863 | 0.175 |  | 0.138 | 0.711 |  | 3.083 | 0.082 |  | 0.134 | 0.715 |  | 2.795 | 0.097 |
|  |  | Alpha-diversity index | | 0.004 | 0.949 |  | 0.560 | 0.456 |  | 1.230 | 0.269 |  | 0.027 | 0.869 |  | 1.325 | 0.252 |  | 0.719 | 0.398 |  | 0.067 | 0.796 |
|  |  | Nest ID |  | 1.518 | **0.020** |  | 2.081 | **<.001** |  | 2.425 | **<.001** |  | 2.402 | **<.001** |  | 5.803 | **<.001** |  |  |  |  | 1.412 | **0.045** |
|  | *PD Faith* |  | Model | Adj. R^2^ = 0.165 | |  | Adj. R^2^ = 0.320 | |  | Adj. R^2^ = 0.364 | |  | Adj. R^2^ = 0.336 | |  | Adj. R^2^ = 0.643 | |  | Adj. R^2^ = -0.002 | |  | Adj. R^2^ = 0.124 | |
|  |  |  |  | 1.538 | 0.016 |  | 2.278 | <.001 |  | 2.559 | <.001 |  | 2.380 | <.001 |  | 5.897 | <.001 |  | 0.776 | 0.462 |  | 1.384 | 0.053 |
|  |  | Intercept |  | 2.229 | 0.138 |  | 0.505 | 0.479 |  | 3.857 | 0.051 |  | 0.038 | 0.847 |  | 0.421 | 0.517 |  | 1.409 | 0.237 |  | 0.015 | 0.901 |
|  |  | Sibling relatedness | | 0.021 | 0.886 |  | 1.225 | 0.270 |  | 2.167 | 0.144 |  | 0.168 | 0.683 |  | 2.628 | 0.107 |  | 0.149 | 0.700 |  | 2.727 | 0.101 |
|  |  | Alpha-diversity index | | 2.326 | 0.130 |  | 0.735 | 0.393 |  | 3.909 | 0.050 |  | 0.006 | 0.936 |  | 0.585 | 0.446 |  | 1.486 | 0.224 |  | 0.002 | 0.963 |
|  |  | Nest ID |  | 1.571 | **0.013** |  | 1.995 | **<.001** |  | 2.487 | **<.001** |  | 2.379 | **<.001** |  | 5.524 | **<.001** |  |  |  |  | 1.399 | **0.050** |
